# SUMOylation of MFF coordinates fission complexes to promote stress-induced mitochondrial fragmentation

**DOI:** 10.1126/sciadv.adq6223

**Published:** 2024-10-04

**Authors:** Richard Seager, Nitheyaa Shree Ramesh, Stephen Cross, Chun Guo, Kevin A. Wilkinson, Jeremy M. Henley

**Affiliations:** ^1^ School of Biochemistry, University of Bristol, Biomedical Sciences Building, Bristol BS8 1TD, UK.; ^2^ Wolfson Bioimaging Facility, Faculty of Life Sciences, University of Bristol, Bristol BS8 1TD, UK.; ^3^ School of Biosciences, University of Sheffield, Alfred Denny Building, Sheffield, S10 2TN, UK.; ^4^ School of Physiology, Pharmacology and Neuroscience, University of Bristol, Biomedical Sciences Building, Bristol BS8 1TD, UK.

## Abstract

Mitochondria undergo fragmentation in response to bioenergetic stress, mediated by dynamin-related protein 1 (DRP1) recruitment to the mitochondria. The major pro-fission DRP1 receptor is mitochondrial fission factor (MFF), and mitochondrial dynamics proteins of 49 and 51 kilodaltons (MiD49/51), which can sequester inactive DRP1. Together, they form a trimeric DRP1-MiD-MFF complex. Adenosine monophosphate–activated protein kinase (AMPK)–mediated phosphorylation of MFF is necessary for mitochondrial fragmentation, but the molecular mechanisms are unclear. Here, we identify MFF as a target of small ubiquitin-like modifier (SUMO) at Lys^151^, MFF SUMOylation is enhanced following AMPK-mediated phosphorylation and that MFF SUMOylation regulates the level of MiD binding to MFF. The mitochondrial stressor carbonyl cyanide 3-chlorophenylhydrazone (CCCP) promotes MFF SUMOylation and mitochondrial fragmentation. However, CCCP-induced fragmentation is impaired in MFF-knockout mouse embryonic fibroblasts expressing non-SUMOylatable MFF K151R. These data suggest that the AMPK-MFF SUMOylation axis dynamically controls stress-induced mitochondrial fragmentation by regulating the levels of MiD in trimeric fission complexes.

## Teaser

A key molecular step is identified for the fragmentation and removal of damaged mitochondria that is essential for cell health.

## INTRODUCTION

Mitochondria form interconnected networks that undergo continuous cycles of fusion and fission to govern mitochondrial morphology and function. Under basal conditions, the overall morphology does not change, with balanced fusion and fission events (*1*, *2*). Fusion is necessary to maintain mtDNA copy number, respiration capacity, and membrane potential (*3*) and also helps to compensate for defects in mitochondrial function by fusing healthy mitochondria with suboptimal functioning mitochondria (*4*). Mitochondrial fission, on the other hand, ensures equal distribution of mitochondria during cell division (*5*), generates sufficiently small mitochondria for transport around the cell to sites of energy demand, particularly important in polarized cells such as neurons (*6*, *7*), and is also crucial for quality control, to isolate damaged mitochondria for removal by mitophagy (*2*, *8*, *9*).

The architecture of the mitochondrial network responds dynamically to fluctuating bioenergetic demands and cellular stress (*10*–*12*). Moderate cell stress, such as nutrient deprivation, ultraviolet (UV)–C irradiation, or cycloheximide exposure, increases fusion [termed stress-induced mitochondrial hyperfusion (SIMH)], which elongates mitochondria, promotes mitochondrial function, and is cytoprotective (*13*–*15*). Conversely, during mitochondrial dysfunction or severe cellular stress, such as oxidative stress or treatment with mitochondrial inhibitors, the mitochondrial network undergoes fragmentation (*16*–*18*), which results in reduced mitochondrial function and increased mitophagy and is associated with apoptosis (*9*, *16*, *19*, *20*). Thus, the balance of mitochondrial fusion/fission is fundamental for mitochondrial function and cellular homeostasis, and dysregulation of these systems is a prominent feature in multiple diseases (*21*).

Fission is mediated by recruitment of dynamin-related protein 1 (DRP1) from the cytosol to the mitochondrial outer membrane, where it oligomerizes and powers membrane scission via guanosine 5′-triphosphate (GTP) hydrolysis (*22*, *23*). There are four known DRP1 receptors: mitochondrial fission 1 protein (Fis1), mitochondrial fission factor (MFF), and mitochondrial dynamics proteins of 49 and 51 kDa (MiD49 and MiD51, respectively), each of which can independently recruit DRP1 to the mitochondrial surface (*16*, *24*, *25*). MFF is the main pro-fission receptor, and its overexpression fragments mitochondria, whereas knockdown results in severe mitochondrial elongation (*16*, *26*). Although MiD49/51 can recruit DRP1 (*25*, *27*, *28*), their effect on fission is less clear because their overexpression not only increases DRP1 recruitment but also causes mitochondrial elongation (*25*, *28*, *29*), likely due to sequestering inactive DRP1 (*16*, *28*). MiD receptors bind to a broad range of DRP1 oligomeric states, including DRP1 mutants with impaired guanosine triphosphatase (GTPase) activity or lacking the ability to form higher oligomeric states (*30*). In contrast, it has been reported that MFF favors binding to higher-order DRP1 oligomers and has significantly impaired ability to interact with mutants lacking GTPase activity or the capacity to oligomerize (*30*, *31*). Although Fis1 has important roles in mitophagy and asymmetric mitochondrial division (*32*–*34*), it plays a relatively minor role in fission (*24*, *26*).

DRP1, MiD, and MFF form a trimeric complex in which MiD proteins facilitate the MFF-DRP1 interaction (*35*). Moreover, MiD51 can inhibit MFF-induced activation of DRP1 GTPase activity (*24*). Thus, the MiD proteins serve as a platform for DRP1 recruitment and assembly, and the differential association of mitochondrially bound DRP1 with the receptors determines fission (*30*). However, how this trimeric complex adapts rates of mitochondrial fission to meet fluctuating bioenergetic demands and mitochondrial stress remains poorly understood.

Adenosine monophosphate–activated protein kinase (AMPK) is a stress response kinase that maintains energy homeostasis. During times of enhanced energy expenditure (signaled by an increase in the adenosine monophosphate/adenosine triphosphate ratio), AMPK is activated to promote energy-producing processes while minimizing energy-demanding processes (*36*). AMPK phosphorylates MFF at Ser^155^ and Ser^172^ in response to mitochondrial stress (*18*, *37*), a modification that is necessary and sufficient to promote mitochondrial fission (*18*) and has been shown to occur during mitophagy (*38*). However, how this phosphorylation event regulates the fission machinery at a molecular level is an important unanswered question.

The posttranslational modifier protein small ubiquitin-like modifier (SUMO) is reversibly conjugated to target proteins to regulate their functions, interactions, and activity. Sentrin-specific proteases (SENP1-3 and SENP5-7) regulate the deconjugation of SUMO from targets. SUMOylation generally occurs within the consensus sequence ψK*x*D/E, where ψ is a large hydrophobic residue and *x* is any amino acid (*39*, *40*). The major SUMO isoforms are SUMO1-3, with SUMO2 and SUMO3 forming poly-SUMO chains, often terminated by SUMO1 (*41*, *42*). Mitochondrial protein SUMOylation regulates mitochondrial morphology and function (*43*, *44*). For example, SUMO1-ylation stabilizes DRP1, promotes fission (*43*, *45*), and has roles in apoptosis (*44*), whereas DRP1 SUMO2/3-ylation reduces binding to MFF and inhibits cytochrome c release and cell death (*46*, *47*).

Here, we identify MFF as an important SUMO target and demonstrate that AMPK-mediated phosphorylation of MFF enhances SUMOylation during times of mitochondrial stress. MFF phosphorylation/SUMOylation does not increase DRP1 binding per se but remodels the DRP1-MiD-MFF fission complex by displacing the inhibitory MiD receptors, thus facilitating stress-induced mitochondrial fission. These findings establish a link between MFF phosphorylation and the molecular events that govern fission and identify MFF SUMOylation as a crucial step in coupling bioenergetic stress to dynamic regulation of mitochondrial morphology.

## RESULTS

### MFF is SUMOylated at Lys^151^ by SUMO1 and SUMO2/3

**Fig. 1.**
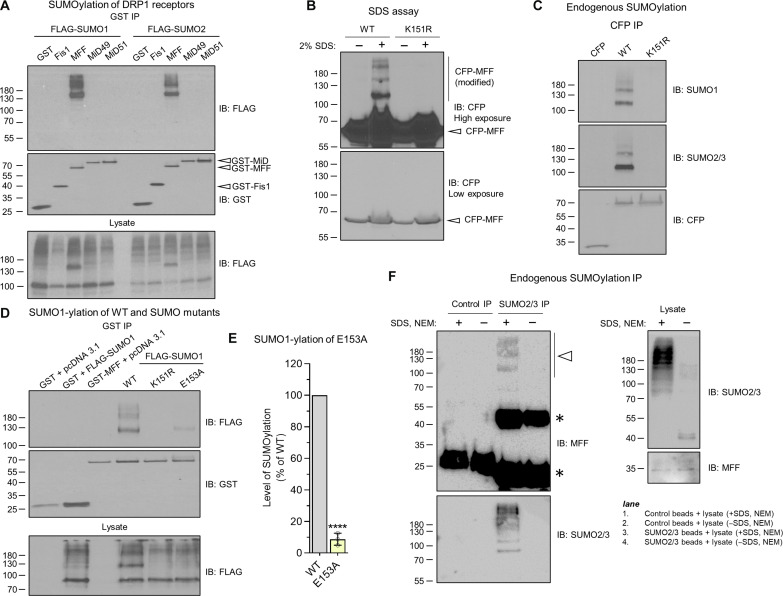
MFF is poly-SUMOylated at Lys^151^. (**A**) HEK293T cells were co-transfected with GST-tagged DRP1 receptors [Fis1 (mouse), MFF (human, isoform 1), and MiD49 or MiD51 (mouse)] and either FLAG-SUMO1 or FLAG-SUMO2. GST immunoprecipitates and lysate were immunoblotted for FLAG and GST. (**B**) Wild-type (WT) or K151R cyan fluorescent protein (CFP)–MFF transfected cells were lysed in buffer ± 2% SDS and probed for CFP. (**C**) Blot of endogenously SUMOylated MFF. CFP-MFF (WT or K151R) immunoprecipitates from HEK293T cells were probed for endogenous SUMO1 or SUMO2/3. Note that, in experiments, when FLAG-SUMO is expressed, the mono-SUMOylated species of MFF resolves at ~130 kDa, whereas, when probing for endogenous SUMO, this corresponds to the ~115-kDa band, due to the lack of tag on SUMO. (**D**) Analysis of MFF SUMOylation-deficient mutants. GST pulldowns of the indicated mutants were blotted for FLAG and GST. (**E**) Quantification of SUMO-deficient MFF mutants. *n* = 3, ****P < 0.001, one-sample *t* test. (**F**) SUMO2/3 immunoprecipitation from HEK293T cell lysate, probed for MFF. Lanes 1 and 2 are control lanes using protein G beads. Lanes 3 and 4 are SUMO2/3-enriched samples using anti–SUMO2/3-conjugated beads. HEK293T cells were lysed in buffer ± 4% SDS and 20 mM *N*-ethylmaleimide (NEM) to preserve or inhibit SENP activity in the lysate. Four percent SDS was then diluted to 0.1% in lysis buffer before incubation with beads. Enrichment of SUMO2/3-conjugated proteins in lane 3 was confirmed by SUMO2/3 reprobe (bottom left blot), and deconjugation of SUMO2/3 was confirmed in the lysate blot. Arrowhead indicates endogenous SUMOylated MFF, and asterisk indicates the nonspecific antibody bands.

DRP1 SUMOylation plays key roles in its recruitment to mitochondria and regulation of morphology (*43*–*47*). Therefore, we wondered whether these processes were also regulated by SUMOylation of the DRP1 receptors. To test this, we transfected human embryonic kidney (HEK) 293T cells with FLAG-SUMO and glutathione *S*-transferase (GST)–DRP1 receptors. Following pulldowns and blotting for FLAG, we observed a prominent band at ~130 kDa and, above this, a robust ladder of SUMO1 and SUMO2 conjugation to MFF (Fig. 1A and fig. S1A), indicative of the presence of mono-SUMOylated MFF and poly-SUMO chains, respectively. SUMO is an ~11 kDa protein, and GST-MFF is ~64 kDa, and so SUMOylated MFF resolves higher than expected. This is a known phenomenon, attributable to the position of SUMOylation within the target protein, causing some proteins to resolve slower by SDS–polyacrylamide gel electrophoresis (PAGE) and affecting the apparent molecular weight (*48*). No FLAG signal was detected for the other receptors, indicating that MFF is likely the only DRP1 receptor SUMOylated under these conditions.

The highly conserved sequence ^150^LKRE^153^ (corresponding to Lys^151^ in human MFF isoform 1 and Lys^125^ in isoforms 2 to 5) conforms to the SUMO consensus motif and was identified as a candidate SUMOylation site in MFF (fig. S1, B and C). Lys^151^ was mutated to a non-SUMOylatable arginine (K151R) and MFF SUMOylation investigated by expressing wild-type (WT) or K151R cyan fluorescent protein (CFP)–MFF in HEK293T cells followed by blotting for CFP after lysis under strong denaturing conditions (±2% SDS, to retain SUMO conjugation by impairing the activity of deSUMOylating enzymes). The band-shifted modified forms of CFP-MFF disappear for MFF-WT when SDS is absent and are not present in either condition with the MFF-K151R mutant (Fig. 1B). We then probed CFP-MFF-WT and K151R immunoprecipitates for endogenous SUMO1 or SUMO2/3 and observed a complete absence of SUMOylation in the K151R mutant (Fig. 1C). Last, mutation of the adjacent Glu^153^ residue in CFP-MFF to disrupt the SUMO consensus sequence while retaining the modifiable lysine also results in a severe decrease in SUMOylation (Fig. 1, D and E). Together, these experiments identify Lys^151^ as the sole SUMOylation site in MFF.

We next validated SUMOylation of endogenous MFF. To do this, we used SUMO2/3 antibody–conjugated beads to immunoprecipitate endogenous SUMO2/3 conjugates from HEK293T cells, followed by immunoblotting for MFF. As a negative control, we lysed cells in the absence of SDS and *N*-ethylmaleimide (NEM) to retain endogenous SENP activity in the lysate to facilitate deSUMOylation (Fig. 1F). SUMOylated proteins were enriched in the SUMO2/3 immunoprecipitates, and a MFF immunoreactive ladder of high–molecular weight species, similar in pattern to those observed in Fig. 1 (B and C), was detected only in the SUMO2/3 immunoprecipitate samples lysed in the presence of SDS/NEM. This MFF immunoreactive band was absent when cells were lysed in the absence of SDS and NEM, confirming that MFF is endogenously SUMOylated (Fig. 1F).

We have previously reported that MFF is polyubiquitinated (*49*). To determine whether mixed SUMO and ubiquitin chains are present on MFF, we used recombinant SENP1 or ubiquitin-specific protease 2 (USP2) to selectively deSUMOylate or deubiquitinate, respectively, WT CFP-MFF immunoprecipitated from HEK293T. SENP1 treatment removed SUMO but had no effect on ubiquitin, and USP2 treatment removed ubiquitin from MFF without removing SUMO. These data indicate that these two modifications are independent and that there are no mixed SUMO-ubiquitin chains on MFF (fig. S1D).

### MFF SUMOylation is not required for basal DRP1 recruitment or mitochondrial morphology

**Fig. 2.**
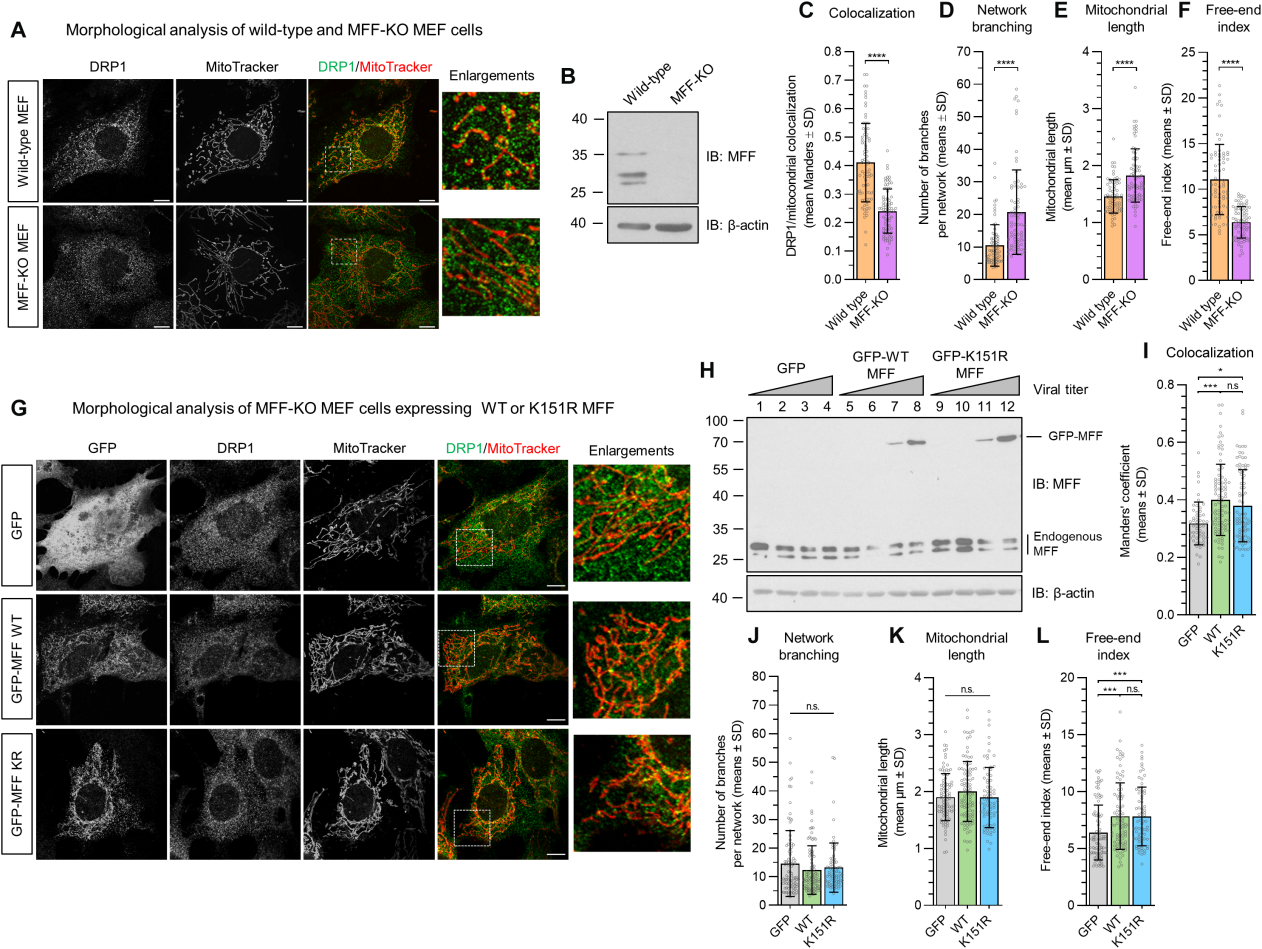
Mitochondrial morphology analysis and DRP1 recruitment in MFF-KO MEF cells expressing MFF-WT or MFF-K151R. (**A**) Confocal images of WT and MFF–knockout (KO) MEF cells stained for DRP1 and mitochondria using MitoTracker. Enlargements show zoomed section of the highlighted area. Scale bars, 10 μm. (**B**) MEF cell lysate probed for MFF. MFF-KO cells lack all detectable isoforms of MFF. (**C**) Manders’ colocalization quantification of DRP1 and MitoTracker, *n* = 3, 84 to 98 cells were imaged. (**D** to **F**) Mitochondrial morphology analysis of MEF WT and MFF-KO cells. (D) number of branches per network, (E) mean mitochondrial length, and (F) free-end index. Mann-Whitney test was used to determine significance, 70 to 74 cells were imaged from three independent experiments, ****P < 0.0001. (**G**) Confocal images of MFF-KO MEF cells expressing either GFP, GFP-MFF-WT, or MFF-K151R. Enlargements show zoomed section of the highlighted area. Scale bars, 10 μm. (**H**) Viral titers of GFP, GFP-MFF-WT, or K151R infection of WT MEF cells were used to determine appropriate viral amount to infect cells with. The volume in lanes 8 and 12 were used for subsequent experiments. (**I**) Manders’ colocalization of DRP1 and MitoTracker [GFP, *n* = 2, 52 cells; and GFP-MFF (WT and K151R), *n* = 3, 85 to 91 cells]. (**J** to **L**) Mitochondrial morphology analysis of MFF-KO cells expressing GFP, WT-MFF, or K151R-MFF. (J) Network branching, (K) mitochondrial length, and (L) free-end index. *n* = 3, 73 to 95 cells, *P < 0.05 and ***P < 0.0005, Kruskal-Wallis test followed by Dunn’s multiple comparisons test. n.s., not significant.

As reported previously (*16*), MFF–knockout (KO) mouse embryonic fibroblast (MEF) cells (lacking all isoforms of MFF) exhibit an elongated and fused mitochondrial network (Fig. 2, A and B). There was also a decrease in mitochondrially associated DRP1 in MFF-KO cells (Fig. 2C). Moreover, quantification of the mitochondrial network showed an increase in the number of branches and average mitochondrial length in the MFF-KO cells (Fig. 2, D and E) and a reduction in the free-end index (a parameter to determine the extent of fragmentation; Fig. 2F).

To define the role of MFF SUMOylation in basal mitochondrial morphology, we used lentiviruses to express green fluorescent protein (GFP)–tagged MFF-WT or K151R in MFF-KO cells. Mitochondria were labeled with MitoTracker and stained for DRP1 (Fig. 2G). Viral titers were adjusted to ensure expression of MFF constructs at approximately endogenous levels (Fig. 2H, note that lanes 8 and 12 express at similar levels to each other and to endogenous MFF, so these titers were used in all subsequent experiments).

Both MFF-WT and K151R localized to mitochondria and rescued DRP1 recruitment to a similar extent (Manders’ values = 0.40 and 0.38, respectively; Fig. 2I) and at levels similar to the WT MEF cells (Manders’ value = 0.41; Fig. 2C). Quantitative analysis of mitochondrial morphology showed no differences in network branching (Fig. 2J) or mitochondrial length (Fig. 2K) between GFP, GFP-MFF-WT, and K151R.

We did, however, detect an increase in the free-end index for both MFF-WT and K151R-MFF above the GFP control, but there was no difference between WT and non-SUMOylatable MFF [6.4% (GFP) versus 7.8% (MFF-WT and K151R); Fig. 2L]. These data show that viral expression of WT or MFF-K151R in MFF-KO cells can at least partially rescue the fission defects and indicate that MFF SUMOylation is not required for basal DRP1 recruitment. Furthermore, preventing MFF SUMOylation had no discernible effects on mitochondrial architecture under basal conditions.

### Phosphorylation at Ser^155^ and Ser^172^ promotes MFF SUMOylation

**Fig. 3.**
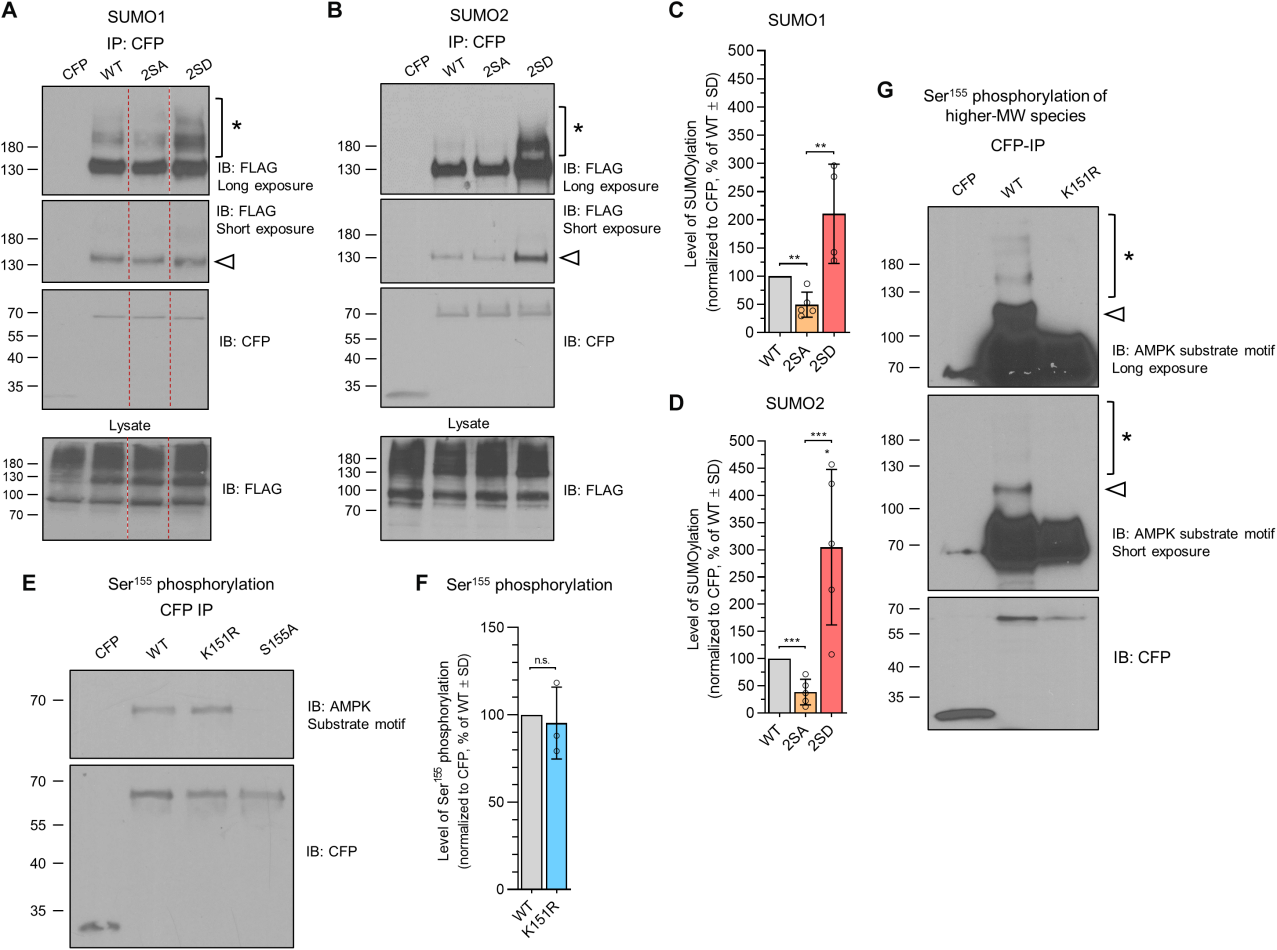
MFF SUMOylation is promoted by AMPK-mediated phosphorylation. (**A** and **B**) SUMOylation of MFF phosphorylation mutants. HEK293T cells were co-transfected with CFP-tagged 2SA/D MFF mutants and either (A) FLAG-SUMO1 or (B) FLAG-SUMO2. Immunoprecipitates and lysates were blotted for FLAG and CFP. Blot was cropped from larger blot (fig. S2E). Arrowhead indicates mono-SUMOylated MFF band, and asterisk shows higher–molecular weight bands. (**C** and **D**) Quantification of SUMOylation of MFF phosphorylation mutants. One-sample *t* test was performed to determine significance between mutants and WT, and unpaired *t* test was performed to determine significance between mutants, *n* = 4 or 5; *P < 0.05, **P < 0.01, and ***P < 0.005. (**E**) Ser^155^ phosphorylation of MFF-K151R. HEK293T cells were transfected with the indicated CFP-MFF mutants, and immunoprecipitates were blotted for Ser^155^ phosphorylation using an AMPK substrate motif antibody. S155A mutant was used to confirm specificity of the antibody. (**F**) Quantification of phosphorylation state of MFF-K151R, *n* = 3, one-sample *t* test. (**G**) Ser^155^ phosphorylation of MFF-WT and K151R. HEK293T cells were transfected with CFP-MFF (WT or K151R), and immunoprecipitates were blotted for Ser^155^ phosphorylation. Arrowhead corresponds to the band similar to the size of the mono-SUMOylated MFF species, and asterisk represents higher–molecular weight (MW) species.

In response to bioenergetic stress, AMPK phosphorylates MFF at Ser^155^ and Ser^172^, leading to mitochondrial fragmentation (*18*, *37*, *38*). Ser^155^ lies within a phosphorylation-dependent SUMO consensus motif [Ψ-K-*x*-E-*x*-(*x*)-S; fig. S1C], which is a strong predictor of positive regulation of SUMOylation by phosphorylation (*50*). Therefore, we co-expressed either double phospho-null (S155A/S172A) MFF-2SA or phospho-mimetic (S155D/S172D) MFF-2SD mutants with FLAG-SUMO1 or FLAG-SUMO2 in HEK293T cells and assessed their SUMOylation. MFF-2SD was significantly more SUMOylated than MFF-2SA (Fig. 3, A to D), with the 2SA mutant exhibiting significantly less SUMOylation than WT MFF. Similar results were observed for individual analysis of both the mono- and poly-SUMOylated forms of MFF (fig. S2, A to D).

We next measured Ser^155^ phosphorylation of MFF-WT and K151R using an AMPK substrate motif antibody. No signal was detected in the S155A mutant, confirming that the antibody is specific for this phosphorylation site of MFF (Fig. 3E). Both MFF-WT and K151R had comparable levels of Ser^155^ phosphorylation (Fig. 3F), indicating that SUMOylation does not affect MFF phosphorylation. Longer exposure of phospho-Ser^155^ blots revealed a ladder of bands for MFF-WT but not for K151R (Fig. 3G), similar in pattern to that observed for SUMOylated MFF. Moreover, the band representing mono-SUMOylated MFF (Fig. 1, B and C) and MFF phosphorylation (Fig. 3G) correspond at ~115 kDa, demonstrating the existence of SUMOylated phospho-MFF. Together, these data indicate that AMPK-mediated phosphorylation promotes MFF SUMOylation and that these modifications occur concurrently.

The mitochondrial-anchored protein ligase (MAPL) and the deSUMOylating enzymes SENP3 and SENP5 regulate DRP1 SUMOylation and mitochondrial morphology (*43*, *44*, *46*, *47*, *51*). Consistent with these reports, we observed that small interfering RNA (siRNA)–mediated knockdown of MAPL reduced, whereas SENP3 and SENP5 knockdown enhanced, MFF SUMOylation (fig. S2, F and G). These findings indicate that a complex array of proteins regulate the SUMOylation status of MFF, promoted by MAPL and AMPK, and antagonized by SENP3/5.

### DRP1 binding to MFF is independent of MFF phosphorylation and SUMOylation

**Fig. 4.**
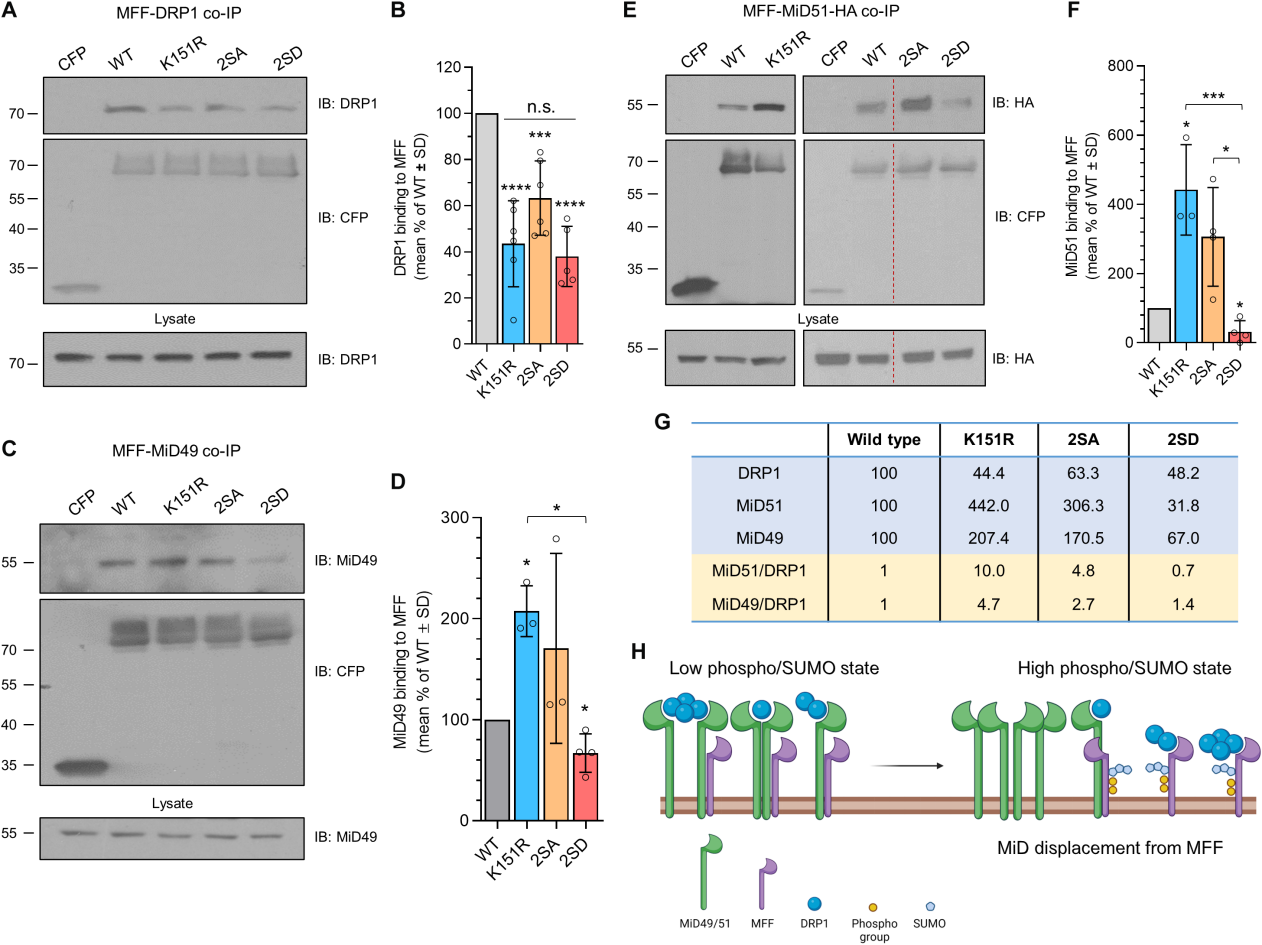
MFF posttranslational modifications regulate the MiD/DRP1 ratio in the fission complex. (**A**) Representative blot of endogenous DRP1 binding to CFP-MFF mutants. HEK293T cells were transfected with the indicated CFP-MFF mutants, and immunoprecipitates were probed for DRP1. (**B**) Quantification of DRP1 binding, *n* = 5 or 6. Representative blot of (**C**) endogenous MiD49 and (**E**) MiD51-HA binding to MFF mutants. Immunoprecipitates from HEK293T were co-transfected with MiD-HA, and the indicated CFP-MFF mutants were probed for HA. Uncropped blot of E in fig. S3D. (**D** and **F**) Quantification of (D) MiD49 and (F) MiD51-HA binding to MFF mutants, *n* = 3 or 4, *P < 0.05, ***P < 0.005, and ****P < 0.001, one-sample *t* test used to determine significance from WT; one-way analysis of variance (ANOVA) was used to determine significance between groups. (**G**) The table shows the relative amounts of DRP1 and MiD within the different MFF complexes, compared to WT, obtained from the quantifications [(B), (D), and (F)]. The MiD-to-DRP1 ratio is highlighted in yellow, calculated from the values in blue. (**H**) Schematic of DRP1-MiD-MFF rearrangement in response to MFF phosphorylation and SUMOylation. Created with BioRender.com.

AMPK-mediated phosphorylation of MFF during bioenergetic stress drives mitochondrial fission (*18*). Extrapolating our data that phosphorylation promotes MFF SUMOylation, we hypothesized that the enhanced SUMOylation of MFF-2SD would increase DRP1 binding, whereas the diminished SUMOylation of MFF-2SA or the complete lack of SUMOylation of MFF-K151R would reduce DRP1 binding. To test this hypothesis, we probed immunoprecipitates of CFP-MFF mutants from transfected HEK293T cells for endogenous DRP1 (Fig. 4, A and B, and fig. S3A). Unexpectedly, there was no difference in DRP1 binding between the MFF-2SD and MFF-2SA mutants, and DRP1 binding for each of the MFF mutants was significantly reduced compared to MFF-WT (Fig. 4, A and B). The data for MFF-K151R and MFF-2SA are consistent with a model of MFF SUMOylation promoting fission. However, because MFF-2SD and MFF-2SA mutants have been reported to have opposing effects on fission (*18*), their similar binding to DRP1 was unexpected. Thus, we interpret these results to indicate that these regulatory processes are more complex than a simple linear sequence of MFF phosphorylation, leading to MFF SUMOylation that, in turn, promotes MFF binding to DRP1.

### SUMOylated MFF displaces MiD from the fission complex

We wondered whether the similar binding of DRP1 to MFF-2SD and MFF-2SA could be explained by a mechanism in which MFF phosphorylation does not enhance DRP1 binding per se. Rather, we hypothesized that the stoichiometry of the DRP1-MiD-MFF trimeric fission complex (*35*) might be altered by MFF phosphorylation. To test this, we measured endogenous MiD49 and MiD51-HA binding to MFF-WT, MFF-K151R, MFF-2SA and MFF-2SD under basal conditions. Both MiD49 and MiD51 bound significantly more to MFF-K151R and significantly less to MFF-2SD compared to MFF-WT (Fig. 4, C to F). Furthermore, MiD51-HA bound significantly more to MFF-2SA than MFF-2SD (Fig. 4, E and F), and a similar trend was also detected for MiD49-HA binding (fig. S3, B and C). In addition, both the MiD-HA proteins and endogenous MiD49 bound significantly more to MFF-K151R compared to MFF-2SD (Fig. 4, C to F, and fig. S3, B and C).

We next quantified the relative ratios of MiD49/51 to DRP1 in DRP1-MiD-MFF complexes (Fig. 4G). The ratio of MiD51 to DRP was ~10-fold greater in complexes containing non-SUMOylatable MFF-K151R compared to that in complexes containing MFF-WT. Moreover, in MFF-2SA containing complexes, the MiD51/DRP1 ratio was ~4.8-fold greater than that in complexes containing MFF-WT, whereas the MiD51/DRP1 ratio in complexes containing MFF-2SD complex was ~30% less than that in complexes containing MFF-WT. Likewise, the relative ratio of MiD49/DRP1 in non-SUMOylatable MFF-K151R complexes was ~4.7-fold more than that in WT, and the ratio in the MFF-2SA and MFF-2SD containing complexes was 2.7 and 1.4, respectively. These results indicate that, rather than directly promoting MFF-DRP1 binding, the interplay between MFF phosphorylation and SUMOylation has a more nuanced effect by modulating the stoichiometry of the DRP1-MiD-MFF fission complex (Fig. 4H).

Multiple oligomeric states of DRP1 can bind MiD49/51 (*30*). Because our co-IP data (Fig. 4A) do not distinguish between potentially different oligomeric states, we carried out cross-linking experiments using the non-cleavable cross-linker dextran sulfate sodium (DSS) before co-IP of DRP1 with MFF mutants (fig. S3E). Fission complexes containing monomeric and higher-order states of DRP1 were detected, confirming that MFF-WT can bind to multiple oligomeric states of DRP1. There was no notable difference between MFF mutants binding to monomeric versus oligomeric DRP1 (fig. S3F). These results suggest that posttranslationally modified forms of MFF can bind equally well to different oligomeric states of DRP1. Figure 4H is a schematic illustrating the relative differences in MiD association within the DRP1-MiD-MFF complex in response to phosphorylation and SUMOylation. This model illustrates that both reduced and enhanced phosphorylation/SUMOylation forms of MFF can co-immunoprecipitate similar levels of DRP1 but that enhanced MFF phosphorylation/SUMOylation displaces MiD from the trimeric complex.

### CCCP-induced mitochondrial stress enhances MFF SUMOylation and displaces MiD51

**Fig. 5.**
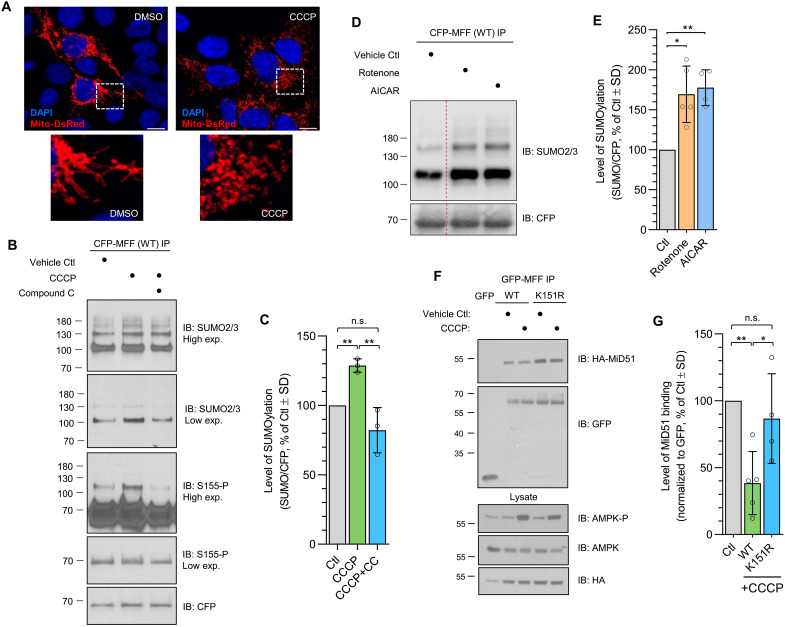
AMPK activation enhances MFF SUMOylation and MiD51 displacement in response to CCCP. (**A**) Confocal images of HEK293T cells transfected with mito-DsRed following 1 hour of treatment with 10 μM CCCP. Scale bars, 10 μm. (**B**) HEK293T cells were transfected with CFP-MFF (WT or K151R) and treated with 10 μM CCCP for 1 hour before lysis, either alone or in combination with the AMPK inhibitor compound C (10 μM). CFP-MFF immunoprecipitates were blotted for SUMO2/3, Ser^155^ phosphorylation (low- and higher-exposure blots are shown), and CFP. (**C**) Quantification of SUMOylation of WT-MFF following 1 hour of CCCP in the presence or absence of compound C. Representative of three independent experiments. One-sample *t* test for conditions versus vehicle control (Ctl), and two-sample *t* test for CCCP versus CCCP + CC conditions. **P < 0.01. (**D** and **E**) HEK293T cells were transfected with CFP-MFF (WT) and treated with rotenone (250 ng/ml) or 1 mM AICAR for 1 hour before lysis. CFP immunoprecipitates were blotted for SUMO2/3 and CFP. Quantification presented in (E), *n* = 5 (rotenone) and *n* = 4 (AICAR). Uncropped blot is shown in fig. S4C. (**F**) HEK293T cells expressing GFP-MFF (WT or K151R) and MiD51-HA were treated with CCCP (10 μM, 1 hour). Co-immunoprecipitates were immunoblotted for HA and GFP. (**G**) Quantification of MiD51-HA binding to MFF following CCCP treatment, *n* = 4 or 5. One-sample *t* test for CCCP conditions versus vehicle controls, and two-sample *t* test for WT versus K151R CCCP conditions. *P < 0.05 and **P < 0.01.

We next investigated the effects of stress on SUMO2/3-conjugation to MFF. We first used the mitochondrial ionophore carbonyl cyanide 3-chlorophenylhydrazone (CCCP), which has been used extensively to investigate mitochondrial fission (*16*, *26*, *28*, *52*) to induce fragmentation (Fig. 5A). CCCP treatment caused an ~30% increase in SUMO2/3-ylation on MFF (Fig. 5, B and C). However, the AMPK inhibitor compound C (also called dorsomorphin) prevented this CCCP-induced increase in MFF SUMOylation, confirming a central role for AMPK (Fig. 5, B and C). Compound C also blocked the CCCP-induced increase in Ser^155^ phosphorylation of higher molecular weight MFF bands, further indicating that MFF can be both phosphorylated and SUMOylated in an AMPK-dependent manner (Fig. 5B). CCCP treatment of MFF-K151R also increased Ser^155^ phosphorylation (fig. S4A), again consistent with MFF phosphorylation occurring upstream of SUMOylation. To confirm that we are looking at the early-stage events of stress-induced mitochondrial fission, 10 μM CCCP treatment did not induce LC3 lipidation in our experiments (fig. S4B), suggesting that mitophagy is not being induced at the CCCP concentration and time point investigated.

To further substantiate the role of AMPK in this pathway, we quantified MFF SUMOylation in response to treatment with the AMPK activator 5-aminoimidazole-4-carboxamide ribonucleoside (AICAR) and the complex I inhibitor rotenone, which have been shown to lead to MFF phosphorylation (*18*). Both AICAR and rotenone significantly enhanced MFF SUMO2/3 conjugation (Fig. 5, D and E), indicating that specific activation of AMPK, either by a mimetic or inhibition of the electron transport chain, is sufficient to enhance MFF SUMOylation.

MiD51 has been reported to inhibit MFF-induced activation of DRP1 GTPase activity (*24*), and so we focused our investigation on MiD51 binding to MFF following CCCP treatment. CCCP significantly reduced MiD51 binding to MFF (Fig. 5, F and G), consistent with the decreased association of the phospho-mimetic MFF-2SD with MiD proteins (Fig. 4, C to F). MiD51 binding to non-SUMOylatable MFF-K151R was not reduced by CCCP treatment. Similar results were observed for endogenous MiD51 (fig. S4D).

A comparison of MiD51 to DRP1 in MFF-WT and MFF-K151R complexes shows that the MiD51/DRP1 ratio is reduced by >70% in the MFF-WT complex following treatment with CCCP, whereas there was no change in the composition of the MFF-K151R complex (fig. S4D). Consistent with the data shown in Fig. 4, these results indicate that mitochondrial stress enhances MFF phosphorylation and SUMOylation and reduces binding to MiD51 in an MFF SUMOylation-dependent manner.

### MFF SUMOylation is not required for DRP1 recruitment but is required for CCCP-induced fragmentation

Our data support a model whereby enhanced MFF SUMOylation in response to AMPK activation promotes fission by displacing inhibitory MiD proteins from the trimeric DRP1-MiD-MFF fission complex (Fig. 4H). We interrogated this model further using WT and MFF-KO MEF cells. Cells were treated with 10 μM CCCP for 1 hour to induce fragmentation, and then the mitochondria were imaged. In agreement with previous reports (*16*), WT MEF cells exhibit extensive fragmentation in response to CCCP, whereas MFF-KO MEF cells were resistant to CCCP-induced fragmentation (fig. S5A). We quantified the extent of fragmentation using the free-end index, which revealed a severe impairment in fragmentation in the MFF-KO cells (fig. S5C). We confirmed that GFP-MFF-WT can induce fission when expressed in the MFF-KO MEF cells, above MFF-KO levels and comparable to WT levels (fig. S5, B and C).

**Fig. 6.**
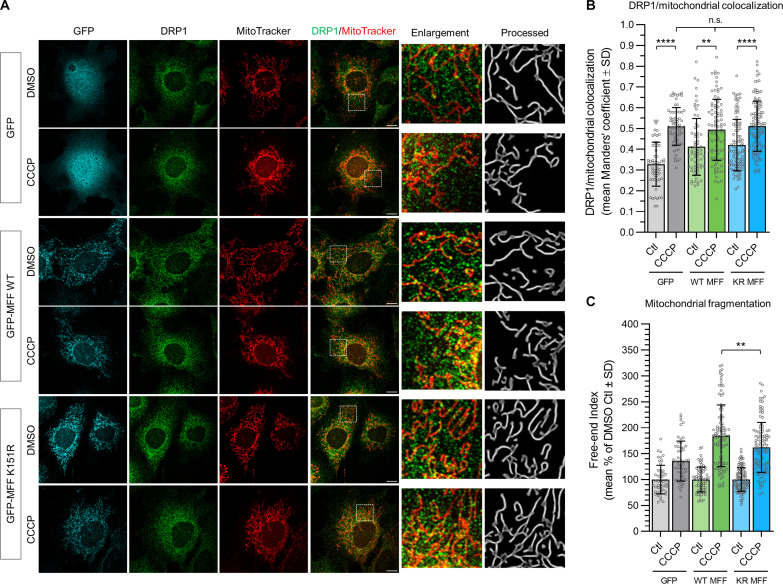
MFF SUMOylation is not necessary for DRP1 recruitment under CCCP treatment but is required for promoting mitochondrial fragmentation. (**A**) Confocal imaging of CCCP-induced mitochondrial fragmentation in MFF-KO MEF cells virally expressing GFP alone, or WT or K151R GFP-MFF. Cells were treated with CCCP (10 μM, 1 hour). Mitochondria were stained using MitoTracker Deep Red, endogenous DRP1 stain is shown in green, and GFP channel is shown in cyan. Processed images of mitochondrial stain with enlargements of highlighted area. Scale bar 10 μm. (**B**) Manders’ colocalization analysis of DRP1 with MitoTracker. Kruskal-Wallis test, 57 to 119 cells were imaged from three independent experiments, **P < 0.01 and ****P < 0.0001. (**C**) Quantification of the free-end index, data were generated from three independent experiments, expressed as percentage of DMSO control, 72 to 110 cells were imaged (for GFP-MFF–expressing cells); two independent experiments, 50 to 53 cells were imaged for GFP-expressing cells. Unpaired *t* test, **P < 0.01.

To investigate the role of MFF SUMOylation in this process, we virally expressed either GFP alone, GFP-MFF-WT, or GFP-MFF-K151R in MFF-KO MEF cells (as in Fig. 2); pretreated with MitoTracker; challenged with 10 μM CCCP for 1 hour; and stained for endogenous DRP1 (Fig. 6A). Colocalization analysis of DRP1 with MitoTracker indicated that both MFF-WT and MFF-K151R recruited equivalent levels of DRP1 to mitochondria following CCCP treatment (Fig. 6B). Moreover, DRP1 recruitment was not impaired in the MFF-KO cells expressing GFP alone, indicating that MFF is not necessary for DRP1 recruitment to mitochondria following CCCP-induced stress.

Quantification of mitochondrial fragmentation following CCCP treatment in the MFF-KO MEF cells revealed that expression of MFF-K151R significantly impaired fragmentation compared to that in MFF-WT–expressing cells (184.4% versus 162.0% for WT and K151R, respectively; Fig. 6C). While CCCP-induced fragmentation in MFF-KO cells expressing GFP-MFF-K151R was higher than that in cells expressing GFP alone (135.6% following CCCP treatment for GFP), these data demonstrate that stress-induced SUMOylation of MFF significantly enhances the fragmentation response. Together, these data reveal that, by rearranging the trimeric DRP1-MiD-MFF complex to displace MiD and promote DRP1-MFF binding, MFF SUMOylation promotes and maximizes stress-induced mitochondrial fission.

## DISCUSSION

**Fig. 7.**
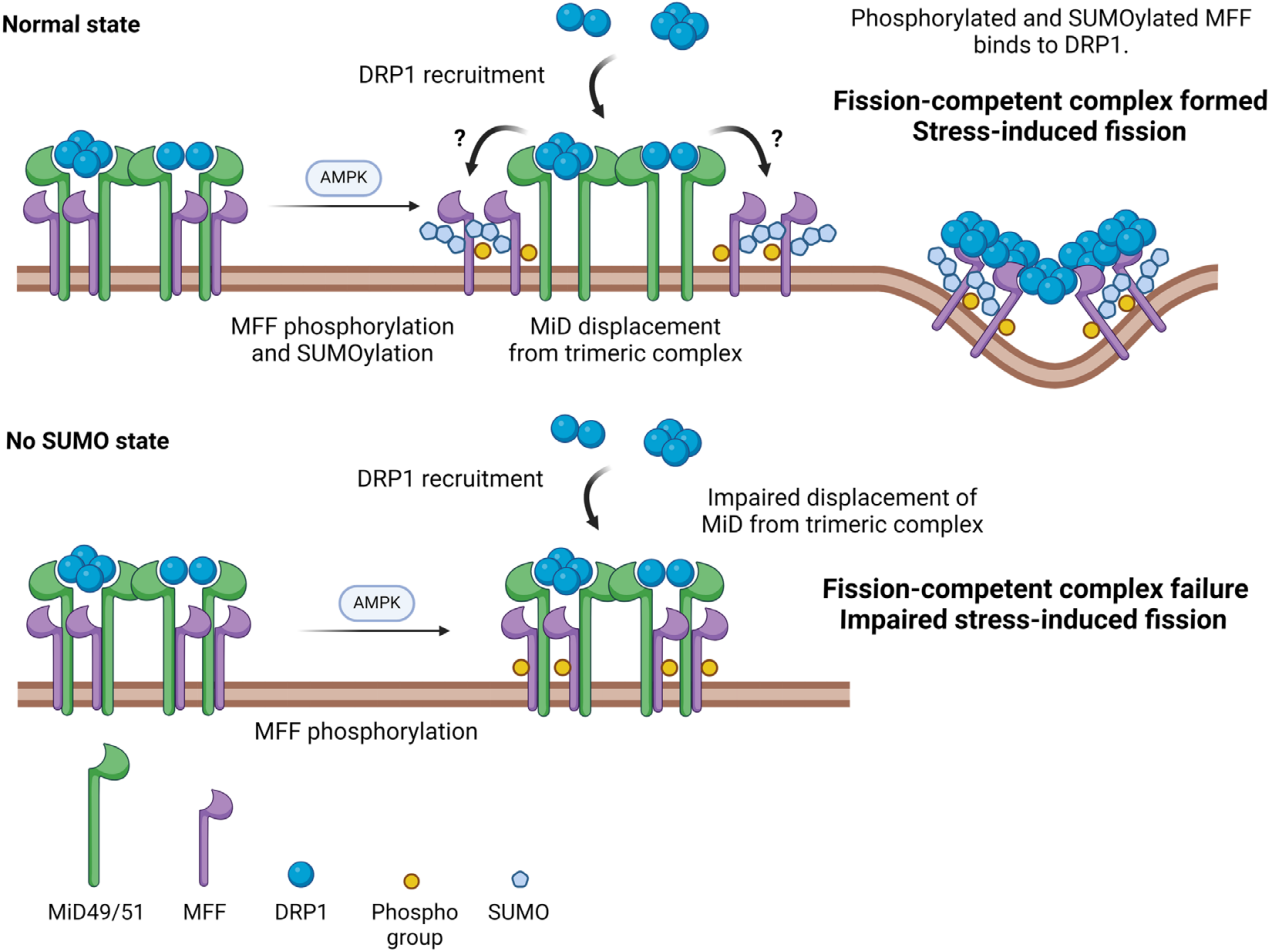
Working model of MFF SUMOylation–dependent stress-induced fission. MFF, DRP1, and MiD49/51 proteins exist in a trimeric complex. Multiple types of DRP1 oligomers exist in the trimeric complex. Upon AMPK activation, MFF is phosphorylated at Ser^155^ and Ser^172^, leading to MFF SUMOylation at Lys^151^ (for simplicity, only one phosphorylation site is shown). In the WT condition, this results in reduced MiD association and displacement from the complex, leading to the formation of MFF-DRP1 fission–competent complexes. How MiD proteins transfer DRP1 to MFF remains to be determined. When MFF cannot be SUMOylated, DRP1 is still recruited, and MFF is still phosphorylated, but MiD proteins remain associated in the trimeric complex. MFF-DRP1 fission complexes are not efficiently formed, leading to impaired fragmentation in response to stress. Created with BioRender.com.

How cells couple mitochondrial dynamics to their bioenergetic state is a fundamental question in cell biology. AMPK-mediated phosphorylation of MFF has been shown to drive mitochondrial fission in response to bioenergetic stress (*18*), but the molecular events underpinning this process have remained elusive. Here, we show that MFF SUMOylation at Lys^151^ plays a key role in stress-induced mitochondrial fission. AMPK-mediated phosphorylation enhances MFF SUMOylation, which promotes mitochondrial fission by displacing the inhibitory MiD proteins from the fission complex. These findings offer a mechanistic explanation of how mitochondrial fission complexes fine-tune the relative ratios of MFF to MiD to dynamically regulate fission under differing conditions (Fig. 7).

We assessed DRP1 recruitment and mitochondrial morphology following reexpression of MFF-WT or MFF-K151R in MFF-KO cells. We show that MFF SUMOylation is not required for DRP1 engagement with the mitochondria or to regulate mitochondrial morphology under basal conditions. These findings are consistent with compensation by other DRP1 receptors that can independently recruit DRP1 and promote fission (*16*, *24*, *25*).

In line with previous reports, we observe that MFF-KO cells exhibit resistance to CCCP induced mitochondrial fragmentation (*16*, *24*, *26*), confirming that MFF is a core component of the fission machinery. However, we show that DRP1 recruitment increases under CCCP conditions in both MFF-WT and MFF-K151R–expressing cells (Fig. 6B), indicating that MFF SUMOylation is not required for DRP1 recruitment under stress. Nonetheless, stress-induced mitochondrial fragmentation is significantly reduced in cells expressing non-SUMOylatable MFF. We interpret these data to indicate that SUMOylation of MFF acts downstream of DRP1 recruitment to promote fragmentation. However, cells expressing non-SUMOylatable MFF-K151R show greater stress-induced mitochondrial fragmentation than MFF-KO cells, suggesting that bioenergetic stress can still induce fragmentation in the absence of MFF, in agreement with previous reports (*16*, *24*). Nonetheless, our data demonstrate that the presence of MFF facilitates and enhances fragmentation and that MFF SUMOylation is required to maximize the fragmentation response. Thus, we propose that MFF SUMOylation provides a mechanism for graduating the severity of stress-induced mitochondrial fragmentation.

Mitochondrial morphology is regulated by balanced fusion and fission (*1*, *2*). Therefore, a fragmented mitochondrial network may be a result of either enhanced fission or reduced fusion. MFF is well established as a pro-fission protein, mediating DRP1-dependent fission (*16*, *26*), which does not negatively regulate fusion (*52*). Thus, our data strongly support a direct role of MFF SUMOylation in mitochondrial fission and not inhibition of fusion.

We show that MFF phosphorylation does not enhance DRP1 association with MFF per se, as has been previously postulated (*18*). Rather, MFF SUMOylation controls the stoichiometry of MiD proteins within the trimeric DRP1-MiD-MFF fission complex. It has been reported previously that MiD proteins can promote CCCP-induced fission in the absence of MFF (*16*, *24*). Our model, however, suggests that, when assembled in the trimeric complex, the MiD proteins act as both a scaffold to assemble DRP1 and a facilitator for the DRP1-MFF interaction. MFF SUMOylation reduces MiD binding, allowing greater binding to DRP1 and activation of fission.

There are likely multiple pathways involved in controlling fission, which can be modulated to ensure nuanced adaptation to a wide range of circumstances. Thus, situations where MiD proteins can promote fission independently of MFF and MiD proteins acting as a scaffold to mediate DRP1-MFF binding are not mutually exclusive. An analogous proposal for fission complex rearrangement has been proposed following UV irradiation (*53*), which suggests that Fis1 binds competitively to MiD51, reducing MiD51-DRP1 binding, and, simultaneously, MFF-DRP1 association is increased to promote fission. It can thus be envisioned that, under different stress conditions, multiple pathways involving different receptors could be activated to elicit appropriate fission responses.

Our data indicate that different oligomeric states of DRP1 can associate with MiD-MFF in the trimeric fission complex and that this is not affected by MFF SUMOylation. These data contrast, in part, with the findings of Yu and colleagues, who reported that MiD proteins can associate with a wide range of active and inactive states of DRP1, whereas MFF favors active forms and higher-order states (*30*). This apparent discrepancy might be due to differences in the time used for DSS cross-linking and/or conditions used to isolate the fission complex. For example, we isolate the trimeric complex containing MiD, which likely explains our detection of a wide range of DRP1 states. Nonetheless, there is close alignment in underlying concepts that MiD proteins act as a scaffold for inactive forms of DRP1 recruited from the cytosol (*28*) and then, in response to fission stimulus, transfer DRP1 to MFF to form an active fission–competent complex (*30*). Precisely how the trimeric complex is rearranged and how DRP1 is transferred from MiD proteins to MFF remain to be established.

In addition to AMPK phosphorylation promoting MFF SUMOylation, we identify both SENP3 and SENP5 as MFF deSUMOylating enzymes (fig. S2F). It has been reported previously that overexpression of SENP5 results in tubulated mitochondria, reduced DRP1 levels, and diminished SUMO conjugation in mitochondrial fractions (*43*). Conversely, SUMO1-ylation increases DRP1 stability and promotes fission (*43*, *45*, *51*) as well as facilitating its oligomerization and localization at mitochondrial fission sites during cell death (*44*).

We have reported previously that DRP1 SUMO2/3-ylation in an oxygen-glucose deprivation ischemia model partitions DRP1 in the cytosol away from the mitochondrial membrane to reduce DRP1-MFF binding (*46*, *47*). This has been proposed as a possible protective pathway to counter ischemia-induced cell death. However, when cells are reoxygenated following ischemia, SENP3 levels rapidly recover leading to DRP1 deSUMOylation, recruitment to mitochondria, and consequent mitochondrial fragmentation and the apoptotic release of cytochrome *c* (*46*, *47*).

DRP1 SUMO1-ylation is dependent on the mitochondrial E3 ligase MAPL, with either MAPL knockdown or overexpression of SENP5 suppressing cytochrome *c* release during apoptosis (*44*). Moreover, MAPL has been reported to regulate mitochondrial quality control and integrity by controlling levels of mitofusin-2 (*54*) and to promote mitophagy via its interaction with GABARAP (*55*). Intriguingly, MAPL functions to maintain endoplasmic reticulum–mitochondrial contact in neurons and restrain mitophagy under mild stress before parkin recruitment to severely damaged mitochondria (*56*).

We have previously reported the ubiquitin E3 ligase parkin plays a role in MFF proteostasis (*49*), and, here, we show that MAPL is a cognate SUMO E3 ligase for MFF (fig. S2G). These findings open the possibility that orchestrated and coordinated ubiquitination and SUMOylation of MFF by parkin and MAPL, respectively, could play key roles in fission, mitophagy, and cell death. We believe that this interplay could dynamically control appropriate mitochondrial responses and represents an important avenue for future work. Furthermore, dissecting the interrelationship between SUMOylation of DRP1 and MFF and how MAPL and SENPs regulate their SUMOylation status to dynamically control appropriate mitochondrial responses should be a focus for future work.

Posttranslational modifications of mitochondrial proteins play key roles in regulating mitochondrial fission during cell division. During mitosis, protein kinase D (PKD) phosphorylates MFF at Ser^155^, Ser^172^, and Ser^275^, modifications necessary and sufficient for mitochondrial fission and correct chromosome segregation during cell division (*57*). This process is independent of AMPK phosphorylation, indicating that MFF has at least two different kinases that act on the same sites during distinct cellular processes. These findings raise the interesting question of whether the PKD-MFF and AMPK-MFF pathways promote mitochondrial fission under different cellular conditions through SUMOylation of MFF.

We show that MFF is modified by both SUMO1 and SUMO2/3 and exhibits multiple lengths of SUMO chains. A prototypic poly-SUMO substate is the promyelocytic leukemia (PML) protein, an important component of PML nuclear bodies (*41*). PML SUMOylation is essential for correct nuclear body localization and formation (*58*, *59*), with distinct SUMO-interacting proteins recruited to the poly-SUMO chain on PML (*60*, *61*), which exhibit a binding preference depending on the SUMO chain composition (*62*). This raises the question of whether the multiple SUMOylation states of MFF and the different chain compositions could potentially act to recruit distinct proteins that recognize the SUMO chain and perform different functions. Future research into the composition and potential interactors of the MFF poly-SUMO chain will yield greater understanding of the role of poly-SUMO chains and potential functions beyond fission.

In conclusion, we show that MFF SUMOylation is a critical step in stress-induced mitochondrial fission. We propose a mechanistic model of fission in which MFF SUMOylation modulates the stochiometric composition of the DRP1-MiD-MFF trimeric fission complex to dynamically regulate the fusion/fission balance to rapidly induce fragmentation during bioenergetic stress (Fig. 7).

## MATERIALS AND METHODS

### Reagents and antibodies

Dulbecco’s modified Eagle’s medium (DMEM; Lonza), heat-inactivated fetal bovine serum (FBS; Sigma-Aldrich), penicillin and streptomycin (Gibco), and 0.05% trypsin-EDTA (Gibco). Lipofectamine 2000 was from Thermo Fisher Scientific. Poly-l-lysine (PLL), rotenone [dissolved in dimethyl sulfoxide (DMSO)], carbonyl cyanide *m*-chlorophenylhydrazone (CCCP; dissolved in DMSO), compound C (dorsomorphin, dissolved in DMSO), β-glycerophosphate, Na-pyrophosphate, NEM, EDTA, Triton X-100, and glycerol were from Sigma-Aldrich. AICAR (dissolved in cell culture–grade H_2_O) was from Tocris. DSS was from Thermo Fisher Scientific, prepared fresh in DMSO. Protease inhibitors (complete, EDTA-free, protease inhibitor cocktail tablets) were from Roche. Glutathione sepharose beads were from GE Healthcare Life Sciences, GFP-Trap beads were from ChromoTek, and anti-SUMO2/3 beads were from Cytoskeleton.

For Western blotting, horseradish peroxidase (HRP)–conjugated anti-mouse (raised in goat), anti-goat (raised in rabbit), anti-rabbit (raised in goat), and anti-rat (raised in rabbit) were obtained from Sigma-Aldrich and used at a dilution of 1:10,000. Primary antibodies for Western blotting are listed in table S1. Primary antibodies used for imaging were mouse anti-DRP1 (BD Biosciences, no. 611113, at 1:400) and chicken anti-GFP (Abcam, no. 13970, at 1:1000). Secondary antibodies for imaging were Cy2 anti-chicken and Cy3 anti-mouse (raised in donkey) from Jackson ImmunoResearch and used at 1:400. MitoTracker Deep Red was obtained from Invitrogen (no. M22426), diluted in DMSO, and used at a final concentration of 100 nM.

### Plasmids and siRNA

A pool of siRNA against MAPL (Mul1) was purchased from Dharmacon (ON-TARGETplus human MUL1 siRNA). Control pool of siRNA was from Dharmacon (ON-TARGETplus Non-targeting Pool). Both were dissolved in ribonuclease (RNAse)–free water and used at a final concentration of 20 nM. Human SENP siRNAs were from Sigma-Aldrich (SENP3, ACGUGGACAUCUUCAAUAA; SENP5, AAGUCCACUGGUCUCUCAUUA; and control targeting luciferase, CUUACGCUGAGUACUUCGA) and used at 100 nM. GST-tagged DRP1 receptors have been described previously (*46*). CFP-MFF was constructed by subcloning the MFF human isoform 1 sequence from GST-MFF into the Bam HI/Hind III sites of pECFP-C1. MitoDS Red (pDsRed2-mito) was from Clontech.

3xFLAG-SUMO1 and SUMO2 were produced by polymerase chain reaction (PCR)–based cloning of human SUMO1 or SUMO2 into the Bam HI site of one of the pCMV-Flag series of vectors, using the primers hSUMO1 Bam HI forward (F) (CTCGGATCCATGTCTGACCAGGAGGCAAAA) and hSUMO1 Bam HI reverse (R) (CACGGATCCTAACCCCCCGTTTGTTCCTG) or hSUMO2 Bam HI F (CTCGGATCCATGGCCGACGAAAAGCCCAAG) and hSUMO2 Bam HI R (CACGGATCCTAACCTCCCGTCTGCTGTTG). GFP-Fis1 was produced by PCR-based cloning of rat Fis1 [amplified from p3xFLAG-CMV-10-Fis1, a gift from M. Schrader (University of Exeter, UK)] into the Hind III and Eco RI sites of pEGFP-C3 (Clontech), using the primers rFis1 Hind III F (CTCAAGCTTATGGAAGCCGTGCTGAACGAG) and rFis1 EcoR1 R (GTGGAATTCCCTTCAGGATTTGGACTTGGACAC). Mutants of MFF were generated by KOD Hot Start DNA Polymerase PCR-based site-directed mutagenesis (see table S2 for primers).

Lentiviral GFP-MFF constructs were produced in the plasmid pXLG3-PX-GFP-WPRE. pAcGFP-tagged human MFF isoform 1 was subcloned from pAcGFP-C1-MFF [a gift from G. Voeltz (Addgene, plasmid no. 49153)] by digestion of pAcGFP-C1-MFF with Nhe I and Bam HI to isolate the pAcGFP-MFF insert and ligating into Spe I and Bam HI cut pXLG3-PX-GFP-WPRE in place of the GFP. The MFF-K151R mutant was produced in exactly the same way after first mutating Lys^151^ to arginine in pAc-GFP-C1-MFF, as described below.

### Generation of MiD-HA and MiD-GFP

RNA was extracted from HEK293T cells using QIAGEN RNeasy mini kit as per the manufacturer’s instructions. Briefly, a confluent 6-cm dish of HEK293T cells was scraped into 600 μl of RLT buffer supplemented with β-mercaptoethanol and centrifuged at 16,000 relative centrifugal force (rcf). Supernatant was precipitated using an equal volume of 70% ethanol and transferred to an RNeasy Mini Spin column. Column was washed and RNA eluted into an RNAse-free microcentrifuge tube with 25 μl of RNase-free water. cDNA was synthesized from RNA using a RevertAid First Strand kit (Thermo Fisher Scientific) according to the manufacturer’s protocol.

The primers in table S3 were used to amplify the complete coding sequence of MiD49 and MiD51 from cDNA and subcloned into the Bam H1/Hind III sites of pEGFP-N1 and pcDNA3.1 (for MiD-GFP and MiD-HA, respectively). The fidelity of all constructs was confirmed by DNA sequencing (Eurofins Genomics).

### Cell culture and transfection

HEK293T cells were from the European Collection of Cell Cultures. MEFs (WT and MFF-KO) have been described previously (*16*). HEK293T and MEF cells were cultured in DMEM containing 4 mM l-glutamine supplemented with 10% FBS, streptomycin (100 μg/ml), and penicillin (100 units/ml). Cells were maintained in a humidified atmosphere of 5% CO_2_ at 37°C. For transfection experiments, 6-cm dishes were coated with PLL (0.1 mg/ml). HEK293T cells were seeded at 1.5 × 10^6^ cells per dish in 4 ml of transfection medium (culture medium lacking antibiotics). The following day, HEK293T cells were transfected using Lipofectamine 2000 and incubated for 36-48 hours. siRNA was transfected along with plasmid DNA using Lipofectamine 2000 as above.

### Lentivirus production and transduction

Lentiviral particles were produced in HEK293T cells. Briefly, 2 × 10^6^ cells were seeded into a 6-cm dish. The next day, cells were transfected with 4 μg of pXLG3-based lentiviral plasmid, 3 and 1 μg of the helper plasmids p8.91 and pMD2.G, respectively, using polyethylenimine (Sigma-Aldrich). Transfection mixtures were left on for 4 hours, before replacement with 3 ml of complete DMEM. Culture medium, containing lentiviral particles, was collected 48 hours later, centrifuged at 2500 rcf to pellet cell debris, and filtered through a 0.45-μm syringe filter. Virus-containing supernatant was then aliquoted into 500 μl of aliquots and frozen at −80°C. For cell transduction, lentivirus was thawed, and the desired amount was added dropwisely to the cells being transduced. Transduced cells were passaged several times and used in experiments as appropriate.

### SDS-PAGE and immunoblotting

Polyacrylamide gels (10 to 12%) were made in-house, and samples were resolved by SDS-PAGE. Proteins were transferred to polyvinylidene difluoride membranes (Merck), blocked in 5% milk or 4% bovine serum albumin (BSA; prepared in phosphate-buffered saline with Tween 20 (PBS-T) for 1 hour at room temperature, and incubated with primary antibody for either 1 hour at room temperature or overnight at 4°C (see table S1 for primary antibodies). Membranes were washed with PBS-T and incubated with secondary antibody conjugated to HRP at 1:10,000. Membranes were washed in PBS-T and assayed for chemiluminescence by using enhanced chemiluminesence and x-ray film (Thermo Fisher Scientific) or using a Li-COR Odyssey Fc scanner (Fig. 5D and figs. S3E and S4, B and C).

### Immunoprecipitations and GST pulldowns

For immunoprecipitation experiments, cells were washed in ice-cold PBS and lysed on ice for 45 to 60 min in the following lysis buffer: 20 mM tris (pH 7.4), 137 mM NaCl, 1% Triton X-100, 10% glycerol, 25 mM β-glycerophosphate, 2 mM Na-pyrophosphate, 20 mM NEM, and 2 mM EDTA, supplemented with protease inhibitors. For investigations into covalent modification (Ser^155^ phosphorylation and SUMO/ubiquitin conjugation), lysis buffer was supplemented with 0.1% SDS, and samples were briefly sonicated. For IP experiments in Fig. 5, a lysis buffer of 50 mM tris (pH 7.5), 150 mM NaCl, 1% Triton X-100, 20 mM NEM, protease inhibitors, and 0.1% SDS was used. Lysate was clarified for 20 min at 16,000 rcf at 4°C. Supernatant was collected and kept on ice. Four percent input was taken and 1 volume of 2× Laemmli sample buffer added before heating at 95°C for 10 min. On the remaining lysate, GFP-Trap beads (ChromoTek) were used to perform immunoprecipitations of GFP and CFP-tagged proteins, and glutathione-sepharose 4B beads (GE Healthcare Life Sciences) were used for pulldowns of GST-tagged proteins. Lysate was added to washed beads and incubated at 4°C for 60 min with slow rotation. Beads were washed three times (in lysis buffer without proteases inhibitors, SDS, or NEM). After the final wash, 2× Laemmli buffer was added, and samples were boiled at 95°C for 10 min.

For enrichment of SUMOylated proteins (Fig. 1F), anti–SUMO2/3 antibody–conjugated beads (Cytoskeleton) or control beads (protein G beads, Cytiva) were used according to the manufacturer’s protocol. Briefly, HEK293T cells were lysed in lysis buffer as above supplemented with 4% SDS and 20 mM NEM (or equivalent amount of H_2_O for control conditions). Lysis was initially performed at room temperature for 5 min, and lysate was diluted to 0.1% SDS and then placed on ice for 30 min. Lysate (0.5 mg; 0.5 mg/m) was incubated with 30 μl of beads overnight at 4°C. Beads were washed three times and then boiled in 2× Laemmli buffer.

For chemical cross-linking before co-IP experiments, DSS was used as previously described (*30*), with a slight modification. Briefly, transfected HEK293T cells were washed in PBS (containing 1 mM CaCl_2_ and 0.5 mM MgCl_2_) and incubated with 1 mM DSS at room temperature for 30 min and then quenched in 50 mM tris (pH 7.5) for 15 min at room temperature. Cells were washed in PBS and lysed in 50 mM tris (pH 7.5), 150 mM NaCl, 1% Triton X-100, and 20 mM NEM supplemented with protease inhibitors. Lysate was clarified, and GFP-IP was performed on supernatant as described above. Samples were resolved by SDS-PAGE using a pre-cask 4 to 20% gradient gel (Bio-Rad).

### In vitro deSUMOylation and deubiquitination assay

The catalytically active domain of SENP1 [produced as described previously; (*63*)] and purified USP2 [a gift from the R. Hay lab (University of Dundee, UK)] was used to enzymatically remove SUMO and ubiquitin from MFF, respectively. To obtain sufficient material, multiple 6-cm dishes of HEK293T cells transfected with WT CFP-MFF or CFP (negative control) were washed in PBS and pooled together in lysis buffer containing 0.1% SDS, protease inhibitors, and 20 mM NEM on ice. CFP-MFF was then immunoprecipitated on GFP-Trap beads as per the immunoprecipitation protocol. Beads were washed three times in wash buffer [137 mM NaCl, 50 mM tris (pH 7.4), and 5 mM MgCl_2_] and separated equally into three fresh Eppendorf tubes, and a final concentration of 100 nM GST-SENP1 or 500 nM GST-USP2 was added for 2 hours at 37°C, with occasional agitation (wash buffer added to control and CFP conditions). An equal volume of 2× Laemmli buffer was then added and samples were boiled at 95°C for 10 min.

### Total cell lysis

For cell lysis in Fig. 1B, transfected HEK293T cells were washed in 1× PBS and lysed in the following buffer: 50 mM tris (pH7.4), 137 mM NaCl, 1% Triton X-100, protease inhibitors, and either 2% SDS or equivalent volume of H_2_O. Samples were lysed (initially at room temperature for 5 min and then kept on ice for 30 min), sonicated, an equal volume of 2× Laemmli buffer added, and boiled at 95°C for 10 min. For cell lysis, as in Fig. 2 (B and H), MEF cells were grown in six-well plates, washed in 1× PBS, lysed in 1× Laemmli buffer, and boiled at 95°C for 10 min.

### Densitometry analysis of Western blots

X-ray films were scanned as PNG files and analyzed using ImageJ software. Files were converted to 8-bit format, analyzed using the Gel Analyzer tool and area under the curve values extracted. For immunoprecipitation experiments, all values are normalized to the respective GST or GFP reprobe for unmodified tagged MFF and expressed as a percentage of the control. Corresponding to Fig. 3 (A to D) and fig. S2 (A to D): For investigating SUMO conjugation in CFP-MFF IPs, the mono-SUMO band saturated before the higher–molecular weight bands were detected. Therefore, we took different exposures and performed an independent analysis of the mono-SUMO and the higher–molecular weight bands of SUMOylated MFF. In Fig. 3 (C and D), the SUMO values are mean averages of the mono-SUMO and higher–molecular weight species. In Fig. 5D and figs. S3E and S4 (B and C), blots were developed using a Li-COR Odyssey Fc and quantified using Li-COR Image Studio software. Cropped blots are indicated with a red dotted line (Figs. 3A, 4E, and 5D).

### Immunocytochemistry and imaging

MEF cells were grown on PLL coated glass cover slips and were pretreated with MitoTracker Deep Red at a final concentration of 100 nM for 45 min before fixation. For experiments of CCCP treatment of 1 hour, cells were incubated in MitoTracker dye for 45 min before treatment with CCCP. Cells were fixed in 4% formaldehyde for 15 min. Cells were washed three times in PBS, permeabilized with 0.1% Triton X-100 (in PBS) for 3 to 4 min, and then washed in PBS. Cells were incubated for 3 to 4 min in 100 mM glycine/PBS to quench unreacted formaldehyde and washed once in PBS. To block nonspecific binding, cells were incubated in 3% BSA/PBS for 20 min at room temperature. Primary antibody (DRP1, 1:400; and GFP, 1:1000) was prepared in 3% BSA, and coverslips were incubated with primary antibody for 60 min at room temperature. Coverslips were washed three times with PBS and then incubated with secondary antibody (anti-mouse Cy3 and anti-chicken Cy2, prepared in 3% BSA/PBS at 1:400) for 45 min. Coverslips were washed four times in PBS and mounted on glass microscope slides using Fluoromount-G [containing 4′,6-diamidino-2-phenylindole (DAPI)].

Imaging was carried out using a Leica SP5-II confocal laser scanning microscope attached to a Leica DMI 6000 inverted epifluorescence microscope. Images were captured using a 63× HCX PL APO CS oil-immersion objective, with 512 × 512 pixel resolution and optical zoom of 3× at 400 Hz. *Z*-stacks were taken with 0.25-μm incremental steps. DAPI was excited using a 50-mW 405-nm diode laser, Cy2 was excited using a 150-mW Ar laser (488 nm), Cy3 using a 20-mW solid-state yellow laser (561 nm), and MitoTracker Deep Red using a 20-mW Red He/Ne (633 nm). All the parameters were kept constant for a complete set of experiments.

### DRP1 colocalization analysis

We generated a cytoplasmic mask to designate the area for DRP1-MitoTracker colocalization analysis, which would remove the nuclei and non-cytoplasmic regions, and avoid manually designating the cytoplasmic region, making analysis more objective. Using the DRP1 stain channel, the following workflow was used: (i) Segmentation of the nuclei using the following steps: median filter with a radius of 2 pixels; a global threshold was applied to binarize the image (Otsu method), holes in the binarized image were removed, and nuclei as connected regions to have a two-dimensional (2D) area larger than 50 μm^2^ were identified, using the MorphoLibJ library. (ii) To identify the extent of the cell: median filter applied with a radius of 2 pixels; a global intensity threshold was applied to binarize image (Huang method), and the binarized nuclear and cytoplasmic images were combined to get a complete binary image of the cell; another median filter (2-pixel radius) was applied; fill holes were applied as before, and distance-based watershed transform was used to split any adjacent cells. Cells had a minimum 2D area larger than 400 μm^2^ to be identified. (iii) The nucleus was subtracted from the whole cell to yield the cytoplasmic region. Cytoplasmic objects were identified as before, using connected foreground labeled pixels. Cytoplasm detected area must be larger than 50 μm^2^. The two channels were normalized to the full 8-bit intensity range. Manders’ colocalization was calculated using ImageJ’s coloc2 tool between the DRP1 channel and MitoTracker channel within the cytoplasmic mask.

### Mitochondrial morphology analysis

To analyze the mitochondrial network of MEF cells in an objective and quantifiable manner, we adapted a method developed by Valente and colleagues, who described a macro in conjunction with ImageJ software (*64*). We adjusted the preprocessing steps to better reproduce the mitochondrial morphology of our imaging and manually extracted our own parameters for analysis. First, using the freehand selection tool, we outlined the cell of interest and cleared the outside. The nuclear region was also traced and excluded from analysis. The confocal z-stack was projected to a single image (max intensity), local contrast enhanced (blocksize = 125, histogram bins = 256, and maximum slope = 2), and background subtracted (radius of 10, with sliding paraboloid). Following this, two filters were applied: median filter (radius of 1 pixel) and unsharp filter (sigma radius of 0.4 and mask weight of 0.7). We incorporated a plug-in called Tubeness (sigma of 0.2), which we found increased the detection of smaller and dimmer mitochondria, and also prevented over fragmentation of the network, making the skeleton a more faithful representation of the raw image. Following preprocessing, the image was binarized and skeletonized (ImageJ’s make binary and skeletonize). This generates a 1-pixel outline that allocates three types of pixel, based on the immediate neighbors: End pixels have either one or zero neighbors, slab pixels have two neighbors, whereas junctions have three or four neighbors. We used the ImageJ’s in-built analyze skeleton function, which generates a table of information on the branches and pixels. From the table of branch information, the values were exported to Excel, and various parameters were manually extracted: (i) Mean number of branches per network: mean number of branches within structures containing ≥2 branches. The number of branches column was arranged in numerical order, branches of less than 2 were removed, and the average mean was calculated. (ii) Mean mitochondrial length: extracted from the average branch length, which is the length between two endpoints, an endpoint and junction, or two junctions. The branch length column was arranged in numerical order, nonzero lengths were removed (single pixels), and the average mean was calculated. (iii) Free-end index: number of free ends as a percentage of the total number of pixels detected [sum of free ends/sum of all pixels (free ends, junctions, and slab pixels)] × 100. We used this parameter as a measure of the extent of fragmentation.

### Statistical analysis

Statistical analysis was performed using GraphPad Prism software version 8. For quantification of densitometry of Western blots, all values are presented as means ± SD, expressed as a percentage of control. One-sample *t* test was used to determine significance between conditions and control (set to 100), and unpaired *t* test was used to determine significance between two groups. For multiple comparisons, one-way analysis of variance (ANOVA) was used followed by Tukey’s post hoc test. For analysis of imaging, data were tested for normality distribution using the D’Agostino and Pearson test. If this test was passed, then a parametric test (*t* test and one-way ANOVA followed by Tukey’s post hoc test) was used to determine significance. If failed, then a nonparametric test (Mann-Whitney test and Kruskal-Wallis test followed by Dunn’s post hoc test) was used to determine significance. A *P* value of <0.05 was considered significant. *P* values, independent repeats and statistical approach, are described in the figure legends.

## Supplementary Materials

### This PDF file includes:

Figs. S1 to S5 Tables S1 to S3 References
