# Supplementary material for: SUMOylation of MFF coordinates fission complexes to promote stress-induced mitochondrial fragmentation
Source: Sci Adv. 2024 Oct 4;10(40):eadq6223. doi: 10.1126/sciadv.adq6223 (PMC11451547; doi:10.1126/sciadv.adq6223)
Supplement: Supplementary file 1 — Figs. S1 to S5 Tables S1 to S3 References [file sciadv.adq6223_sm.pdf]

Science Advances
AAAS

Supplementary Materials for

SUMOylation of MFF coordinates fission complexes to promote stress-induced mitochondrial fragmentation

Richard Seager *et al.*

Corresponding author: Kevin A. Wilkinson, kevin.wilkinson@bristol.ac.uk;
Jeremy M. Henley, j.m.henley@bristol.ac.uk

Sci. Adv. **10**, eadq6223 (2024)
DOI: 10.1126/sciadv.adq6223

This PDF file includes:

Figs. S1 to S5
Tables S1 to S3
References

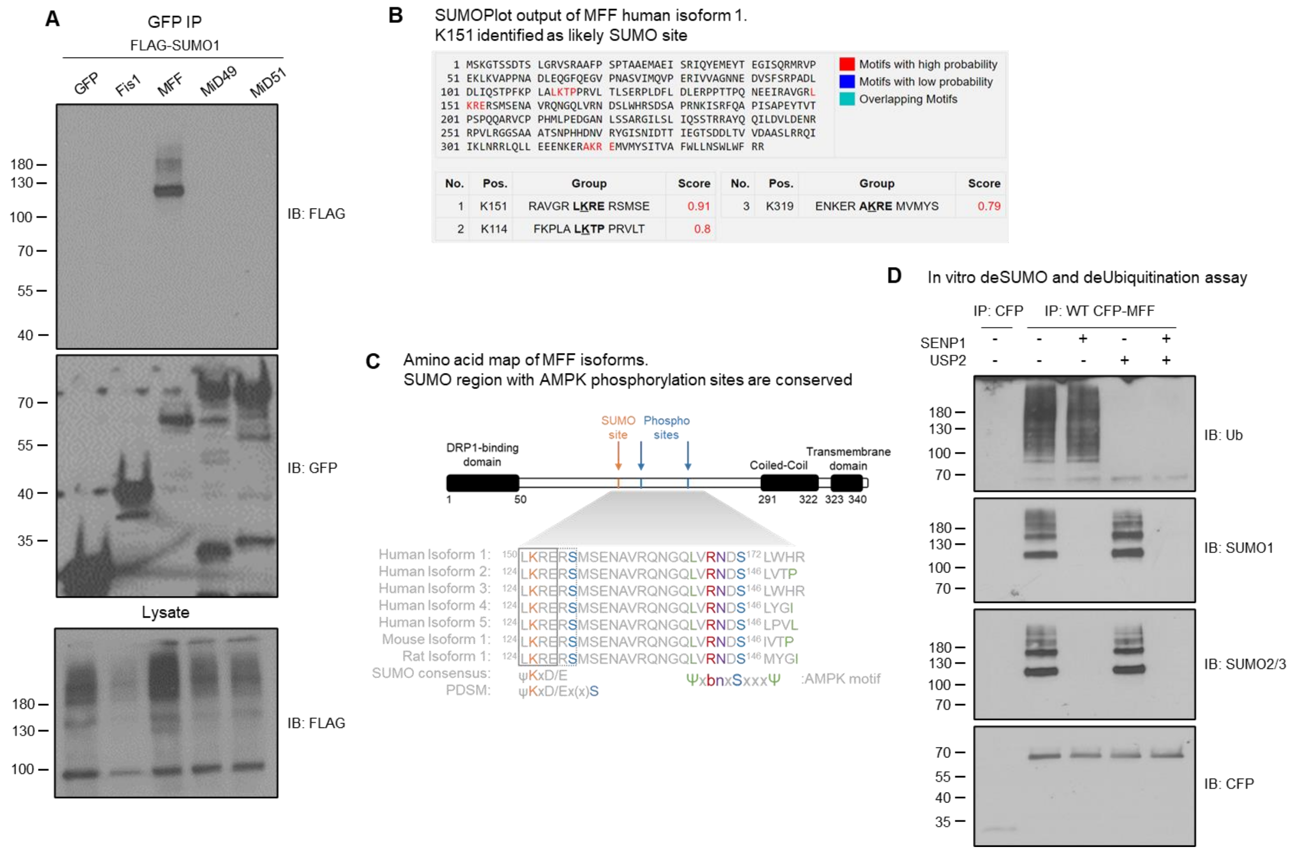

Figure S1. The MFF SUMOylation sequence is well conserved, lies within a PDSM motif, and SUMOylation is independent of MFF ubiquitination. (**A**) Screen of GFP-tagged DRP1 receptors for SUMOylation. HEK293T cells were co-transfected with GFP-Fis1 (rat) GFP-MFF (isoform 1, human), MiD49-GFP or MiD51-GFP (human) and FLAG-SUMO1. GFP-TRAP was used to IP GFP-tagged proteins and immunoprecipitates were blotted for FLAG and GFP. (**B**) SUMOplot^{TM} output for SUMO consensus motifs within the human MFF isoform 1 protein sequence. (**C**) Alignment of human, mouse and rat isoforms of MFF. Schematic representation of MFF, showing the N-terminal DRP1 binding domain, SUMO consensus sequence at ^{150}LKRE^{153} (ψKxD/E, where ψ=hydrophobic amino acid, x=any amino acid) and the phosphorylation sites at Ser^{155} and Ser^{172}, the coiled-coil domain towards the C-terminus, and the single transmembrane domain at the extreme C-terminus. The LKRE motif, as well as the two phosphorylation sites, are conserved among human isoforms 1-5, and the mouse and rat sequences. Due to alternate splicing of human MFF, human isoform 1 has phosphorylation sites at Ser^{155} and Ser^{172}, whereas these correspond to Ser^{129} and Ser^{146} in the other sequences. PDSM=phosphorylation-dependent SUMO consensus motif. The alternative splicing

results in a different amino acid sequence C-terminally to the AMPK site at Ser^{172}. All isoforms contain many of the elements of the AMPK motif ΨxbnxSxxxΨ (Ψ=hydrophobic amino acid, b=basic amino acid, n=neutral amino acid) (*65*). Isoforms correspond to Uniprot entries. (**D**) *In vitro* deSUMOylation and deubiquitination assay of MFF. CFP-MFF (WT) from transfected HEK293T cells was isolated on GFP-TRAP beads. The beads were equally separated into different tubes and treated with 100nM SENP1, 500nM USP2 (or both) for 2hrs at 37°C. Samples were resolved by SDS-PAGE and probed for SUMO1, SUMO2/3 and ubiquitin.

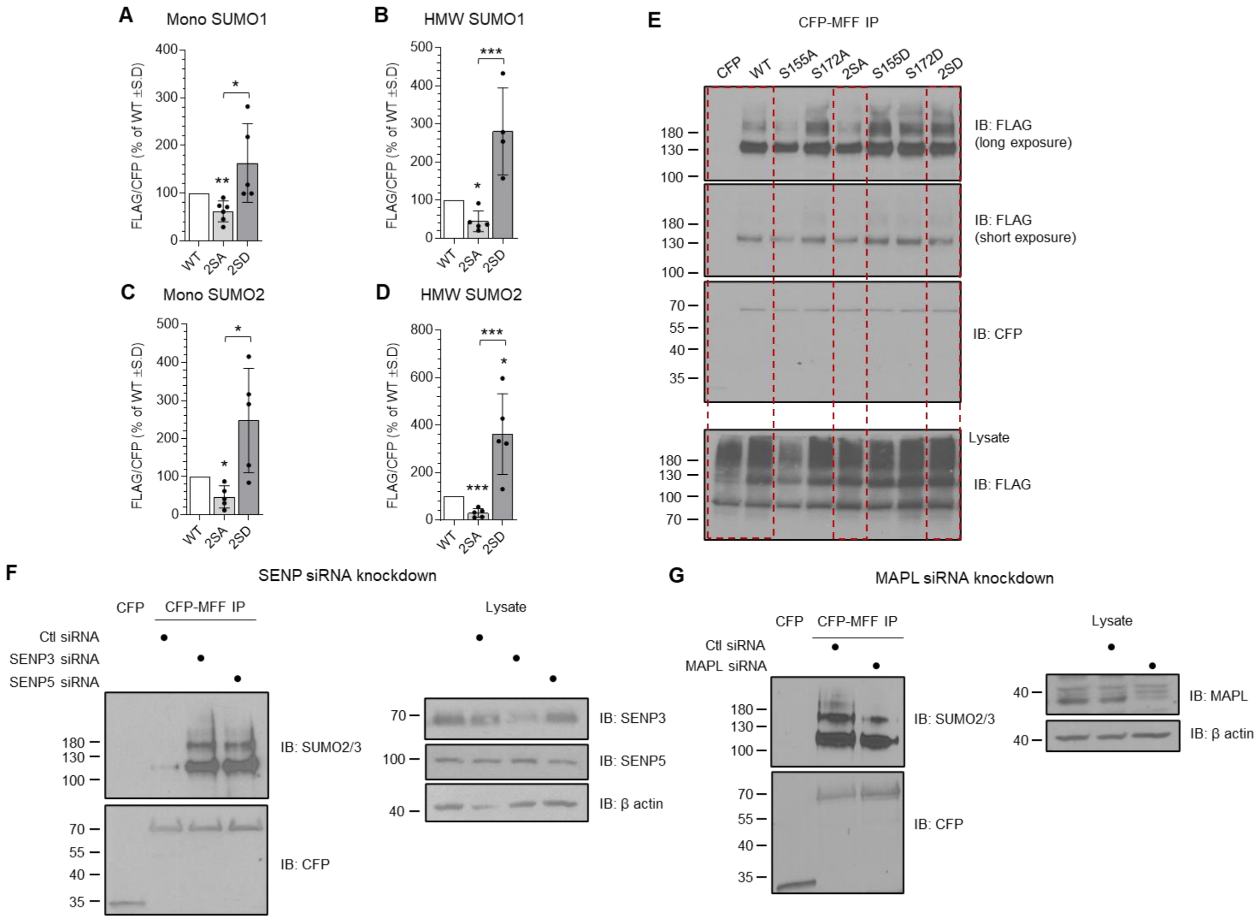

Figure S2. The SUMOylation status of MFF is regulated by AMPK phosphorylation, MAPL, SENP3 and SENP5. (**A-D**) Independent quantification of the mono-SUMO band and the higher molecular weight (HMW) bands of SUMOylated MFF. Corresponds to Fig 3A-D. The mono-SUMO band at 130kDa (**A**) and the higher molecular weight bands (**B**) were quantified, normalised to the CFP blot and expressed as a percentage of wild-type MFF. (**C-D**) As in **A-B** but corresponds to SUMO2. Data generated from 4 (**B**) or 5 (**A, C-D**) independent experiments. One sample t-test used to determine significance between 2SA/D and WT, two sample t-test used to determine significance between 2SA and 2SD. p*<0.05, p**<0.01, p***<0.005. (**E**) SUMOylation of MFF Ser^{155} and Ser^{172} phospho-mutants (showing full blot from Fig 3A). HEK293T cells were co-transfected with the indicated CFP-MFF mutants and FLAG-SUMO1. GFP-TRAP performed on lysate, resolved by SDS-PAGE and probed for FLAG and CFP. (**F**) SENP3/5 deSUMOylates MFF. HEK293T cells were co-transfected with CFP-MFF (WT) and siRNA targeted against SENP3 or SENP5 (100nM, 48hrs). Immunoprecipitates were probed for

SUMO2/3. Lysates probed for SENP3, SENP5 and β-actin. (**G**) MAPL (also called Mul1) is an E3 ligase of MFF. HEK293T were co-transfected with GFP-MFF (WT) and MAPL siRNA at 20nM for 48hrs. Immunoprecipitates were probed for SUMO2/3 and lysate probed for MAPL and β-actin.

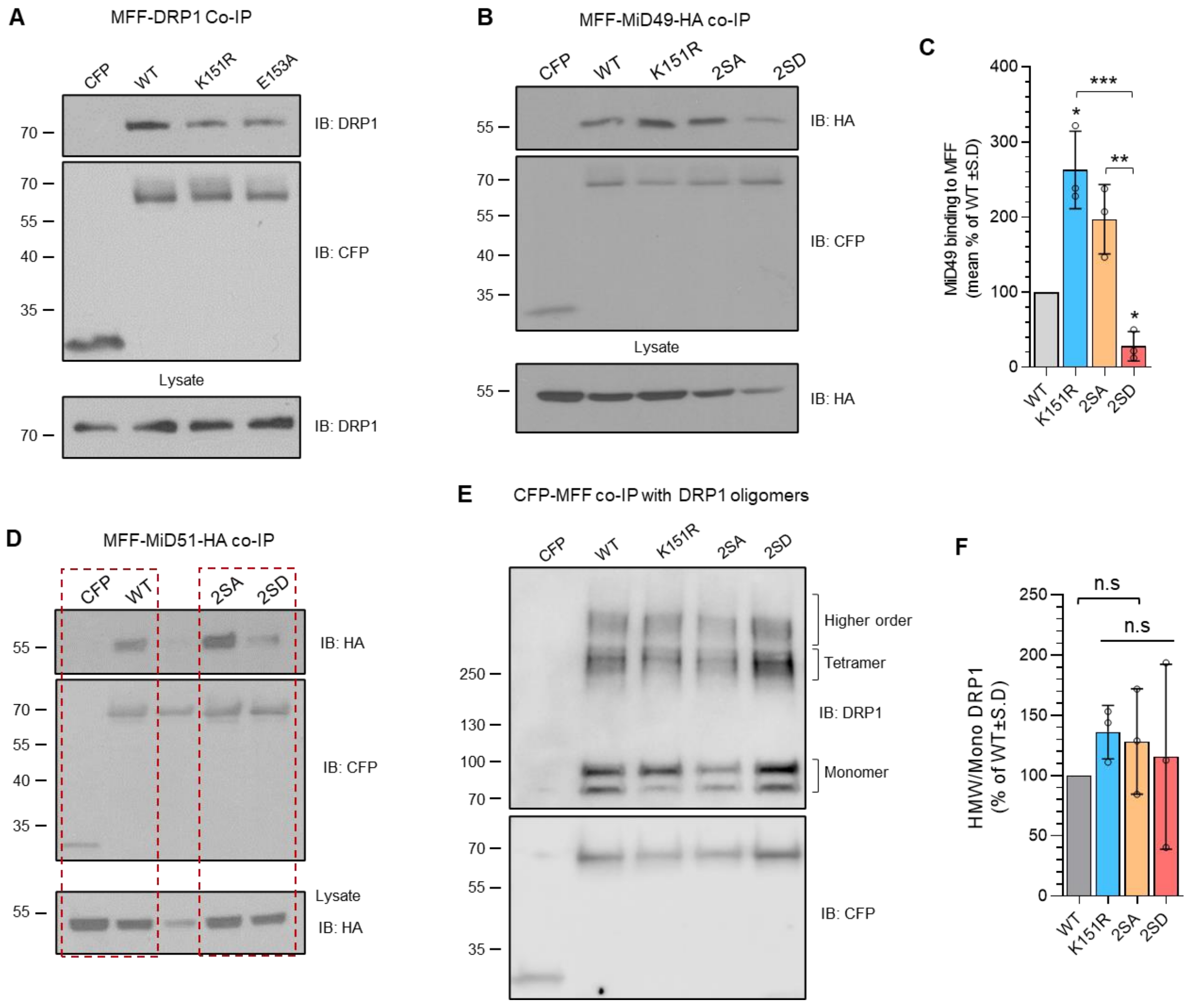

Figure S3. Post-translational modification of MFF alters DRP1 and MiD49 binding, but not binding of different DRP1 oligomeric states. (**A**) Western blot of endogenous DRP1 binding to MFF SUMO mutants. HEK293T cells were transfected with either CFP-MFF (WT, K151R or E153A), co-IP carried out on lysate using GFP-TRAP and samples immunoblotted for endogenous DRP1. (**B**) Western blot of MiD49-HA binding to MFF mutants. HEK293T cells were co-transfected with the indicated CFP-MFF constructs and MiD49-HA. GFP-TRAP was performed to isolate CFP-MFF, resolved by SDS-PAGE and blotted for HA. (**C**) Quantification of MiD49-HA binding to MFF mutants. MiD49 signal was normalised to the CFP reprobe and represented as a percentage of WT±S.D. Data generated from 3 independent experiments, one sample t-test used to determine significance from WT, one-way ANOVA used between groups, p*<0.05, p**<0.01, p***<0.005. (**D**) Uncropped blot for MiD51-HA interaction with MFF mutants, corresponds to Fig 4E. (**E**) HEK293T cells were transfected with the indicated MFF constructs for

48hrs. Prior to lysis, proteins were chemically crosslinked with 1mM DSS for 30 minutes. Cells were then lysed on ice and co-IP performed on samples (as in Figure 4). Samples were resolved on 4-20% gradient gels and probed for DRP1. (**F**) Quantification of the DRP1 oligomers associating with MFF mutants. Tetrameric and higher order states of DRP1 were normalised to monomeric forms of DRP1 and expressed as a percentage of WT. No difference observed between mutants.

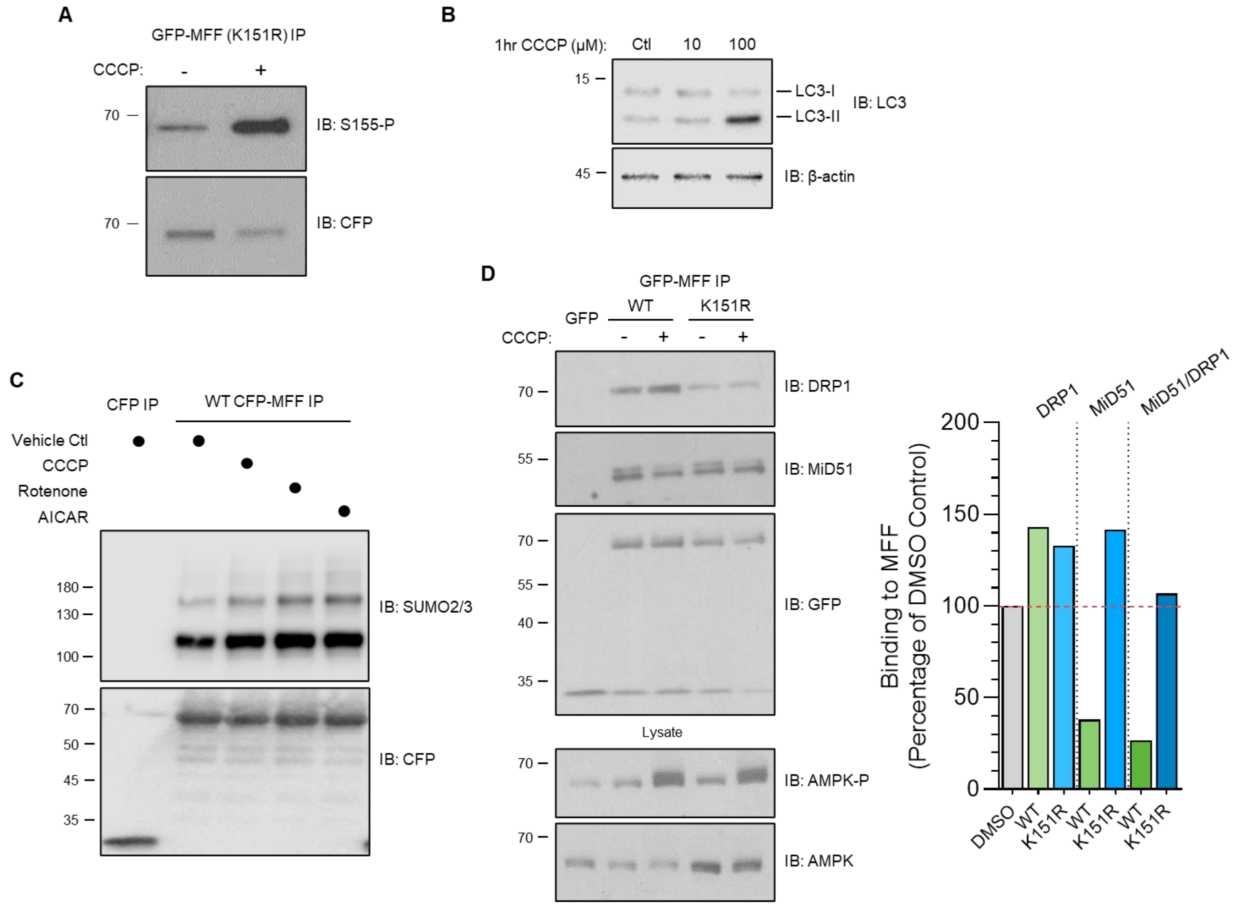

Figure S4. Effects of different mitochondrial stressors on MFF SUMOylation and fission complex composition. (**A**) HEK293T cells were transfected with CFP-MFF-K151R, treated with 10µM CCCP for 1hr, and immunoprecipitates probed for S155 phosphorylation. (**B**) Western blot of HEK293T cells treated with either 10 or 100µM CCCP for 1hr (DMSO as vehicle control). Cells were lysed in Laemmli buffer, boiled and resolved by SDS-PAGE. Blots were probed using LC3A/B and β-actin. Appearance of lower LC3-II band indicates formation of autophagosomes. (**C**) Western blot of CFP-MFF-WT IP from transfected HEK293T treated with CCCP (10µM, 1hr), Rotenone (250ng/mL, 1hr) or AICAR (1mM, 1hr), and immunoprecipitates probed for SUMO2/3. Corresponds to blot in Fig 5D. (**D**) Coimmunoprecipitation experiment from GFP-MFF-WT or K151R transfected HEK293T cells showing binding to endogenous DRP1 and MiD51 following CCCP (10µM, 1hr) treatment. Graph shows binding of DRP1 (column 2 and 3) and MiD51 (column 4 and 5) to MFF (normalised to GFP reprobe) expressed as a percentage of the

corresponding vehicle control, and also shows the change in ratio of MiD51/DRP1 in the WT and K151R fission complex (column 6 and 7).

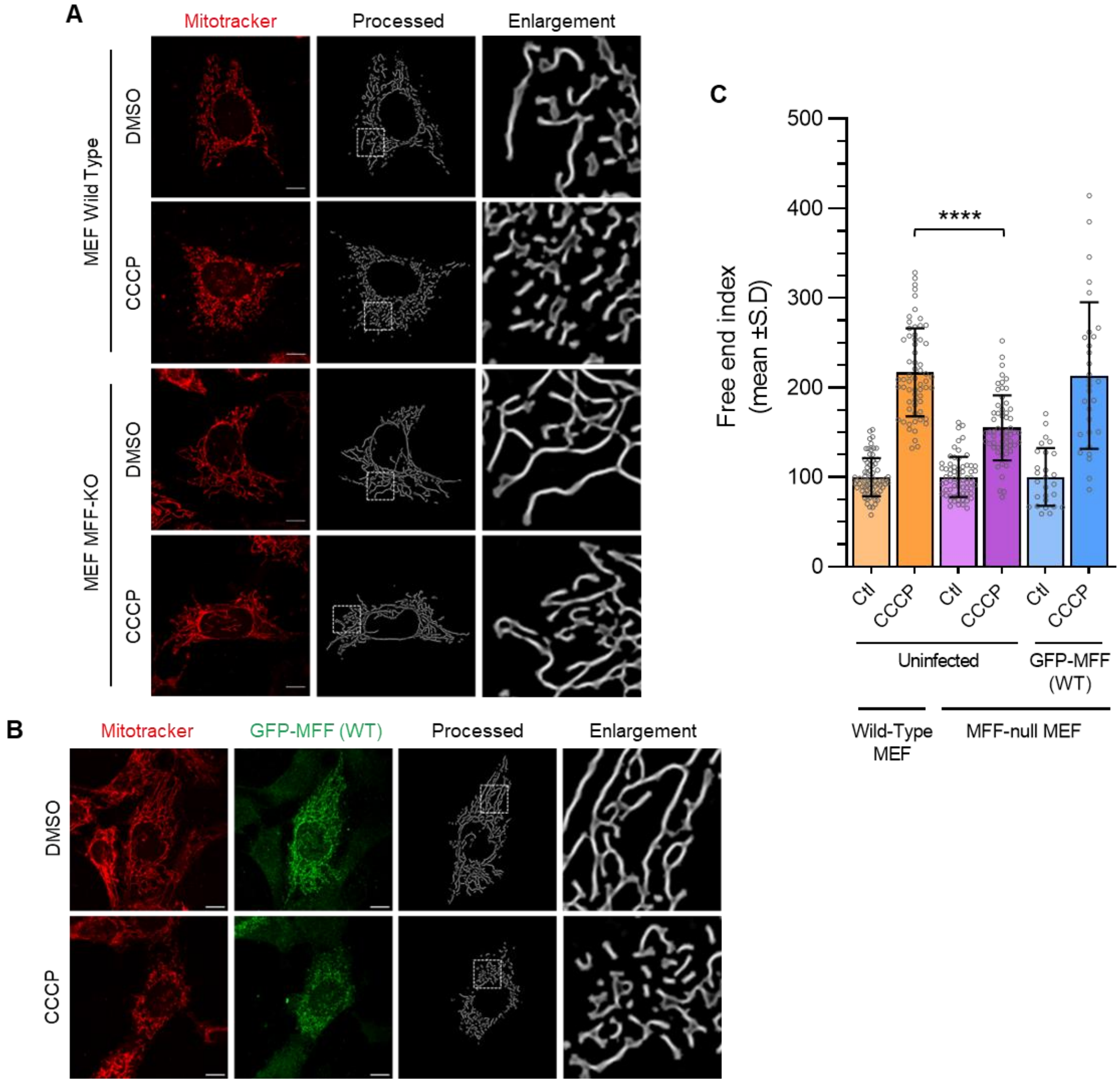

Figure S5. Fragmentation of wild-type MEF and MFF-KO MEF cells with CCCP. (A) Confocal imaging of MEF wild-type and MFF-KO cells treated with CCCP to induce fragmentation. Cells were pretreated with mitotracker before application of 10µM CCCP for 1hr. Confocal images were processed (as described in methods) to generate an outline of the mitochondrial network. Scale bar 10µm. Enlargements show zoomed region of highlighted area. **(B)** GFP-MFF (WT) expression in MEF MFF-KO cells promotes fragmentation following CCCP treatment. Using lenti virus to express GFP-MFF in the MFF-KO MEF cells, mitochondria were stained with mitotracker deep red, and treated with CCCP (10µM, 1hr) before fixing. Red channel shows mitochondrial stain, green channel shows GFP stain to confirm GFP-MFF expression. Scale bar 10µm,

enlargements show zoomed region of highlighted area. **(C)** Quantification of the extent of fragmentation following CCCP treatment of wild-type MEF, MFF-KO MEF cells, and MFF-KO cells expressing GFP-MFF (WT). Values expressed as a percentage of DMSO control, 58-63 cell imaged for wild-type and MFF-KO MEFs, from two experiments. Unpaired t test, p****<0.0001. 27-30 cells imaged for GFP-MFF (WT) expressing cells.

Table S1. List of antibodies, supplier and catalogue numbers used in this study

Protein	Supplier	Reference number	Species	RRID
AMPK α-1	ThermoFisher	AHO1332	Mouse	AB_2536333
P-AMPK (T172)	Cell Signaling	2535	Rabbit	AB_330330
p-(S/T) AMPK substrate motif	Cell Signaling	5759	Rabbit	AB_10949320
DRP1	BD BioScience	611113	Mouse	AB_398424
FLAG	Sigma	F3165	Mouse	AB_259529
GFP	Chromotek	pabg-1-100	Rat	-
GST	GE Healthcare	27457701V	Goat	-
HA	Sigma	H3663	Mouse	AB_262051
LC3A/B	Cell Signaling	4108	Rabbit	-
MiD49	Proteintech	16413-1-AP	Rabbit	AB_2714217
MiD51	Proteintech	20164-1-AP	Rabbit	AB_10639522
MFF	Santa Cruz	SC-398731	Mouse	-
MAPL	Abcam	ab155511	Rabbit	-
SENP3	Cell Signaling	5591	Rabbit	AB_10694546
SENP5	Abcam	ab58420	Rabbit	AB_882487
SUMO1	Cell Signaling	4930	Rabbit	-
SUMO2/3	Cell Signaling	4971	Rabbit	AB_2198425
Ubiquitin	Cell Signaling	3936	Mouse	AB_331292
β-actin	Sigma	A5441	Rabbit	AB_476744

Table S2. Primers for site-directed mutagenesis

Mutation	Primers (5’ to 3’)
K151R	ATCCGAGCAGTTGGCAGACTAAGAAGAGAGCGGTCTATGAGTGAA (For)<br>TTCACTCATAGACCGCTCTCTTCTTAGTCTGCCAACTGCTCGGAT (Rev)
E153A	GCAGTTGGCAGACTAAAAAGAGCGCGGTCTATGAGTGAAAATGCT (For)<br>AGCATTTTCACTCATAGACCGCGCTCTTTTTAGTCTGCCAACTGC (Rev)
S155A	GGCAGACTAAAAAGAGAGCGGGCTATGAGTGAAAATGCTGTTCGC (For)<br>GCGAACAGCATTTTCACTCATAGCCCGCTCTCTTTTTAGTCTGCC (Rev)
S155D	GGCAGACTAAAAAGAGAGCGGGATATGAGTGAAAATGCTGTTCGC (For)<br>GCGAACAGCATTTTCACTCATATCCCGCTCTCTTTTTAGTCTGCC (Rev)
S172A	GGACAGCTGGTCAGAAATGATGCTCTGTGGCACAGATCAGATTC (For)<br>GAATCTGATCTGTGCCACAGAGCATCATTTCTGACCAGCTGTCC (Rev)
S172D	GGACAGCTGGTCAGAAATGATGATCTGTGGCACAGATCAGATTC (For)<br>GAATCTGATCTGTGCCACAGATCATCATTTCTGACCAGCTGTCC (Rev)

Table S3. Primers for generation of MiD49/51 constructs

**Protein/Tag**	**Vector Backbone**	**Primers (5' to 3')**
MiD49/HA	pcDNA3.1	GTGAAGCTTGCCACCATGGCAGAGTTCTCCCAGAAACGG (For)<br>CACGGATCCCTAAGCGTAATCTGGAACATCGTATGGGTAGAGCAGCCCCTCGGGCTCCTGTAG (Rev)
MiD49/GFP	pEGFP-N1	GTGAAGCTTGCCACCATGGCAGAGTTCTCCCAGAAACGG (For)<br>CACGGATCCGCGAGCAGCCCCTCGGGCTCCTGTAG (Rev)
MiD51/HA	pcDNA3.1	GTGAAGCTTGCCACCATGGCAGGCGCTGGTGAGCGCAAA (For)<br>CACGGATCCCTAAGCGTAATCTGGAACATCGTATGGGTACGTCTGCAGCAGCACCTCTGGCTC (Rev)
MiD51/GFP	pEGFP-N1	GTGAAGCTTGCCACCATGGCAGGCGCTGGTGAGCGCAAA (For)<br>CACGGATCCGCCGTCTGCAGCAGCACCTCTGGCTC (Rev)

REFERENCES AND NOTES

1. S. A. Detmer, D. C. Chan, Functions and dysfunctions of mitochondrial dynamics. *Nat. Rev. Mol. Cell Biol.* **8**, 870–879 (2007).

2. G. Twig, A. Elorza, A. J. A. Molina, H. Mohamed, J. D. Wikstrom, G. Walzer, L. Stiles, S. E. Haigh, S. Katz, G. Las, J. Alroy, M. Wu, B. F. Py, J. Yuan, J. T. Deeney, B. E. Corkey, O. S. Shirihai, Fission and selective fusion govern mitochondrial segregation and elimination by autophagy. *EMBO J.* **27**, 433–446 (2008).

3. H. Chen, A. Chomyn, D. C. Chan, Disruption of fusion results in mitochondrial heterogeneity and dysfunction. *J. Biol. Chem.* **280**, 26185–26192 (2005).

4. K. Nakada, K. Inoue, T. Ono, K. Isobe, A. Ogura, Y.-I. Goto, I. Nonaka, J.-I. Hayashi, Inter-mitochondrial complementation: Mitochondria-specific system preventing mice from expression of disease phenotypes by mutant mtDNA. *Nat. Med.* **7**, 934–940 (2001).

5. N. Taguchi, N. Ishihara, A. Jofuku, T. Oka, K. Mihara, Mitotic phosphorylation of dynamin-related GTPase Drp1 participates in mitochondrial fission. *J. Biol. Chem.* **282**, 11521–11529 (2007).

6. M. Cagalinec, D. Safiulina, M. Liiv, J. Liiv, V. Choubey, P. Wareski, V. Veksler, A. Kaasik, Principles of the mitochondrial fusion and fission cycle in neurons. *J. Cell Sci.* **126**, 2187–2197 (2013).

7. T. L. Lewis, S. K. Kwon, A. Lee, R. Shaw, F. Polleux, MFF-dependent mitochondrial fission regulates presynaptic release and axon branching by limiting axonal mitochondria size. *Nat. Commun.* **9**, 5008 (2018).

8. Y. Kageyama, Z. Zhang, R. Roda, M. Fukaya, J. Wakabayashi, N. Wakabayashi, T. W. Kensler, P. H. Reddy, M. Iijima, H. Sesaki, Mitochondrial division ensures the survival of postmitotic neurons by suppressing oxidative damage. *J. Cell Biol.* **197**, 535–551 (2012).

9. Y. Kageyama, M. Hoshijima, K. Seo, D. Bedja, P. Sysa-Shah, S. A. Andrabi, W. Chen, A. Höke, V. L. Dawson, T. M. Dawson, K. Gabrielson, D. A. Kass, M. Iijima, H. Sesaki, Parkin-independent mitophagy requires Drp1 and maintains the integrity of mammalian heart and brain. *EMBO J.* **33**, 2798–2813 (2014).

10. T. Wai, T. Langer, Mitochondrial dynamics and metabolic regulation. *Trends Endocrinol. Metab.* **27**, 105–117 (2016).

11. L. Tilokani, S. Nagashima, V. Paupe, J. Prudent, Mitochondrial dynamics: Overview of molecular mechanisms. *Essays Biochem.* **62**, 341–360 (2018).

12. L. Pernas, L. Scorrano, Mito-morphosis: Mitochondrial fusion, fission, and cristae remodeling as key mediators of cellular function. *Annu. Rev. Physiol.* **78**, 505–531 (2016).

13. L. C. Gomes, G. Di Benedetto, L. Scorrano, During autophagy mitochondria elongate, are spared from degradation and sustain cell viability. *Nat. Cell Biol.* **13**, 589–598 (2011).

14. A. S. Rambold, B. Kostelecky, N. Elia, J. Lippincott-Schwartz, Tubular network formation protects mitochondria from autophagosomal degradation during nutrient starvation. *Proc. Natl. Acad. Sci. U.S.A.* **108**, 10190–10195 (2011).

15. D. Tondera, S. Grandemange, A. Jourdain, M. Karbowski, Y. Mattenberger, S. Herzig, S. Da Cruz, P. Clerc, I. Raschke, C. Merkwirth, S. Ehses, F. Krause, D. C. Chan, C. Alexander, C. Bauer, R. Youle, T. Langer, J.-C. Martinou, SLP-2 is required for stress-induced mitochondrial hyperfusion. *EMBO J.* **28**, 1589–1600 (2009).

16. O. C. Losón, Z. Song, H. Chen, D. C. Chan, Fis1, Mff, MiD49, and MiD51 mediate Drp1 recruitment in mitochondrial fission. *Mol. Biol. Cell* **24**, 659–667 (2013).

17. X. Qi, M. H. Disatnik, N. Shen, R. A. Sobel, D. Mochly-Rosen, Aberrant mitochondrial fission in neurons induced by protein kinase Cδ under oxidative stress conditions in vivo. *Mol. Biol. Cell* **22**, 256–265 (2011).

18. E. Q. Toyama, S. Herzig, J. Courchet, T. L. Lewis, O. C. Loson, K. Hellberg, N. P. Young, H. Chen, F. Polleux, D. C. Chan, R. J. Shaw, AMP-activated protein kinase mediates mitochondrial fission in response to energy stress. *Science* **351**, 275–281 (2016).

19. S. Frank, B. Gaume, E. S. Bergmann-Leitner, W. W. Leitner, E. G. Robert, F. Catez, C. L. Smith, R. J. Youle, The role of dynamin-related protein 1, a mediator of mitochondrial fission, in Apoptosis. *Dev. Cell.* **1**, 515–525 (2001).

20. G. P. Leboucher, Y. C. Tsai, M. Yang, K. C. Shaw, M. Zhou, T. D. Veenstra, M. H. Glickman, A. M. Weissman, Stress-induced phosphorylation and proteasomal degradation of mitofusin 2 facilitates mitochondrial fragmentation and apoptosis. *Mol. Cell* **47**, 547–557 (2012).

21. D. C. Chan, Mitochondrial dynamics and its involvement in disease. *Annu. Rev. Pathol. Mech. Dis.* **15**, 235–259 (2020).

22. J. A. Mears, L. L. Lackner, S. Fang, E. Ingerman, J. Nunnari, J. E. Hinshaw, Conformational changes in Dnm1 support a contractile mechanism for mitochondrial fission. *Nat. Struct. Mol. Biol.* **18**, 20–26 (2011).

23. E. Smirnova, L. Griparic, D. L. Shurland, A. M. Van der Bliek, Dynamin-related protein Drp1 is required for mitochondrial division in mammalian cells. *Mol. Biol. Cell* **12**, 2245–2256 (2001).

24. L. D. Osellame, A. P. Singh, D. A. Stroud, C. S. Palmer, D. Stojanovski, R. Ramachandran, M. T. Ryan, Cooperative and independent roles of the Drp1 adaptors Mff, MiD49 and MiD51 in mitochondrial fission. *J. Cell Sci.* **129**, 2170–2181 (2016).

25. C. S. Palmer, K. D. Elgass, R. G. Parton, L. D. Osellame, D. Stojanovski, M. T. Ryan, Adaptor proteins MiD49 and MiD51 can act independently of Mff and Fis1 in Drp1 recruitment and are specific for mitochondrial fission. *J. Biol. Chem.* **288**, 27584–27593 (2013).

26. H. Otera, C. Wang, M. M. Cleland, K. Setoguchi, S. Yokota, R. J. Youle, K. Mihara, Mff is an essential factor for mitochondrial recruitment of Drp1 during mitochondrial fission in mammalian cells. *J. Cell Biol.* **191**, 1141–1158 (2010).

27. H. Otera, N. Miyata, O. Kuge, K. Mihara, Drp1-dependent mitochondrial fission via MiD49/51 is essential for apoptotic cristae remodeling. *J. Cell Biol.* **212**, 531–544 (2016).

28. C. S. Palmer, L. D. Osellame, D. Laine, O. S. Koutsopoulos, A. E. Frazier, M. T. Ryan, MiD49 and MiD51, new components of the mitochondrial fission machinery. *EMBO Rep.* **12**, 565–573 (2011).

29. J. Zhao, T. Liu, S. Jin, X. Wang, M. Qu, P. Uhlén, N. Tomilin, O. Shupliakov, U. Lendahl, M. Nistér, Human MIEF1 recruits Drp1 to mitochondrial outer membranes and promotes mitochondrial fusion rather than fission. *EMBO J.* **30**, 2762–2778 (2011).

30. R. Yu, S.-B. Jin, M. Ankarcrona, U. Lendahl, M. Nistér, J. Zhao, The molecular assembly state of Drp1 controls its association with the mitochondrial recruitment receptors Mff and MIEF1/2. *Front. Cell Dev. Biol.* **9**, 706687 (2021).

31. R. Liu, D. C. Chan, The mitochondrial fission receptor Mff selectively recruits oligomerized Drp1. *Mol. Biol. Cell* **26**, 4466–4477 (2015).

32. T. Kleele, T. Rey, J. Winter, S. Zaganelli, D. Mahecic, H. P. Lambert, F. P. Ruberto, M. Nemir, T. Wai, T. Pedrazzini, S. Manley, Distinct fission signatures predict mitochondrial degradation or biogenesis. *Nature* **593**, 435–439 (2021).

33. Q. Shen, K. Yamano, B. P. Head, S. Kawajiri, J. T. M. Cheung, C. Wang, J. H. Cho, N. Hattori, R. J. Youle, A. M. Van Der Bliek, Mutations in Fis1 disrupt orderly disposal of defective mitochondria. *Mol. Biol. Cell* **25**, 145–159 (2014).

34. E. Waters, K. A. Wilkinson, A. L. Harding, R. E. Carmichael, D. Robinson, H. E. Colley, C. Guo, The SUMO protease SENP3 regulates mitochondrial autophagy mediated by Fis1. *EMBO Rep*. **23**, e48754 (2022).

35. R. Yu, T. Liu, S.-B. Jin, C. Ning, U. Lendahl, M. Nistér, J. Zhao, MIEF1/2 function as adaptors to recruit Drp1 to mitochondria and regulate the association of Drp1 with Mff. *Sci. Rep.* **7**, 880 (2017).

36. S. Herzig, R. J. Shaw, AMPK: Guardian of metabolism and mitochondrial homeostasis. *Nat. Rev. Mol. Cell Biol.* **19**, 121–135 (2018).

37. S. Ducommun, M. Deak, D. Sumpton, R. J. Ford, A. Núñez Galindo, M. Kussmann, B. Viollet, G. R. Steinberg, M. Foretz, L. Dayon, N. A. Morrice, K. Sakamoto, Motif affinity and mass spectrometry proteomic approach for the discovery of cellular AMPK targets: Identification of mitochondrial fission factor as a new AMPK substrate. *Cell. Signal.* **27**, 978–988 (2015).

38. A. P. Seabright, N. H. F. Fine, J. P. Barlow, S. O. Lord, I. Musa, A. Gray, J. A. Bryant, M. Banzhaf, G. G. Lavery, D. G. Hardie, D. J. Hodson, A. Philp, Y. C. Lai, AMPK activation induces mitophagy and promotes mitochondrial fission while activating TBK1 in a PINK1-Parkin independent manner. *FASEB J.* **34**, 6284–6301 (2020).

39. K. A. Wilkinson, J. M. Henley, Mechanisms, regulation and consequences of protein SUMOylation. *Biochem. J.* **428**, 133–145 (2010).

40. A. Flotho, F. Melchior, Sumoylation: A regulatory protein modification in health and disease. *Annu. Rev. Biochem.* **82**, 357–385 (2013).

41. M. H. Tatham, E. Jaffray, O. A. Vaughan, J. M. P. Desterro, C. H. Botting, J. H. Naismith, R. T. Hay, Polymeric Chains of SUMO-2 and SUMO-3 are conjugated to protein substrates by SAE1/SAE2 and Ubc9. *J. Biol. Chem.* **276**, 35368–35374 (2001).

42. I. Matic, M. van Hagen, J. Schimmel, B. Macek, S. C. Ogg, M. H. Tatham, R. T. Hay, A. I. Lamond, M. Mann, A. C. O. Vertegaal, In vivo identification of human small ubiquitin-like modifier polymerization sites by high accuracy mass spectrometry and an in vitro to in vivo strategy. *Mol. Cell. Proteomics* **7**, 132–144 (2008).

43. R. Zunino, A. Schauss, P. Rippstein, M. Andrade-Navarro, H. M. McBride, The SUMO protease SENP5 is required to maintain mitochondrial morphology and function. *J. Cell Sci.* **120**, 1178–1188 (2007).

44. J. Prudent, R. Zunino, A. Sugiura, S. Mattie, G. C. Shore, H. M. McBride, MAPL SUMOylation of Drp1 stabilizes an ER/mitochondrial platform required for cell death. *Mol. Cell* **59**, 941–955 (2015).

45. Z. Harder, R. Zunino, H. McBride, Sumo1 conjugates mitochondrial substrates and participates in mitochondrial fission. *Curr. Biol.* **14**, 340–345 (2004).

46. C. Guo, K. A. Wilkinson, A. J. Evans, P. P. Rubin, J. M. Henley, SENP3-mediated deSUMOylation of Drp1 facilitates interaction with Mff to promote cell death. *Sci. Rep.* **7**, 43811 (2017).

47. C. Guo, K. L. Hildick, J. Luo, L. Dearden, K. A. Wilkinson, J. M. Henley, SENP3-mediated deSUMOylation of dynamin-related protein 1 promotes cell death following ischaemia. *EMBO J.* **32**, 1514–1528 (2013).

48. M. H. Tatham, M. S. Rodriguez, D. P. Xirodimas, R. T. Hay, Detection of protein SUMOylation in vivo. *Nat. Protoc.* **4**, 1363–1371 (2009).

49. L. Lee, R. Seager, Y. Nakamura, K. A. Wilkinson, J. M. Henley, Parkin-mediated ubiquitination contributes to the constitutive turnover of mitochondrial fission factor (Mff). *PLOS ONE* **14**, e0213116 (2019).

50. V. Hietakangas, J. Anckar, H. A. Blomster, M. Fujimoto, J. J. Palvimo, A. Nakai, L. Sistonen, PDSM, a motif for phosphorylation-dependent SUMO modification. *Proc. Natl. Acad. Sci. U.S.A.* **103**, 45–50 (2006).

51. E. Braschi, R. Zunino, H. M. McBride, MAPL is a new mitochondrial SUMO E3 ligase that regulates mitochondrial fission. *EMBO Rep.* **10**, 748–754 (2009).

52. S. Gandre-Babbe, A. M. van der Bliek, The novel tail-anchored membrane protein Mff controls mitochondrial and peroxisomal fission in mammalian cells. *Mol. Biol. Cell* **19**, 2402–2412 (2008).

53. Z. Zhang, L. Liu, S. Wu, D. Xing, Drp1, Mff, Fis1, and MiD51 are coordinated to mediate mitochondrial fission during UV irradiation-induced apoptosis. *FASEB J.* **30**, 466–476 (2016).

54. J. Yun, R. Puri, H. Yang, M. A. Lizzio, C. Wu, Z.-H. Sheng, M. Guo, MUL1 acts in parallel to the PINK1/parkin pathway in regulating mitofusin and compensates for loss of PINK1/parkin. *eLife* **3**, e01958 (2014).

55. C. T. Ambivero, L. Cilenti, S. Main, A. S. Zervos, Mulan E3 ubiquitin ligase interacts with multiple E2 conjugating enzymes and participates in mitophagy by recruiting GABARAP. *Cell. Signal.* **26**, 2921–2929 (2014).

56. R. Puri, X. T. Cheng, M. Y. Lin, N. Huang, Z. H. Sheng, Mul1 restrains Parkin-mediated mitophagy in mature neurons by maintaining ER-mitochondrial contacts. *Nat. Commun.* **10**, 3645 (2019).

57. E. Pangou, O. Bielska, L. Guerber, S. Schmucker, A. Agote-Arán, T. Ye, Y. Liao, M. Puig-Gamez, E. Grandgirard, C. Kleiss, Y. Liu, E. Compe, Z. Zhang, R. Aebersold, R. Ricci, I. Sumara, A PKD-MFF signaling axis couples mitochondrial fission to mitotic progression. *Cell Rep.* **35**, 109129 (2021).

58. C. Fu, K. Ahmed, H. Ding, X. Ding, J. Lan, Z. Yang, Y. Miao, Y. Zhu, Y. Shi, J. Zhu, H. Huang, X. Yao, Stabilization of PML nuclear localization by conjugation and oligomerization of SUMO-3. *Oncogene* **24**, 5401–5413 (2005).

59. S. Zhong, S. Müller, S. Ronchetti, P. S. Freemont, A. Dejean, P. P. Pandolfi, Role of SUMO-1-modified PML in nuclear body formation. *Blood* **95**, 2748–2752 (2000).

60. Y. Erker, H. Neyret-Kahn, J. S. Seeler, A. Dejean, A. Atfi, L. Levy, Arkadia, a novel SUMO-targeted ubiquitin ligase involved in PML degradation. *Mol. Cell. Biol.* **33**, 2163–2177 (2013).

61. V. Lallemand-Breitenbach, M. Jeanne, S. Benhenda, R. Nasr, M. Lei, L. Peres, J. Zhou, J. Zhu, B. Raught, H. de Thé, Arsenic degrades PML or PML–RARα through a SUMO-triggered RNF4/ubiquitin-mediated pathway. *Nat. Cell Biol.* **10**, 547–555 (2008).

62. A. M. Sriramachandran, K. Meyer-Teschendorf, S. Pabst, H. D. Ulrich, N. H. Gehring, K. Hofmann, G. J. K. Praefcke, R. J. Dohmen, Arkadia/RNF111 is a SUMO-targeted ubiquitin ligase with preference for substrates marked with SUMO1-capped SUMO2/3 chain. *Nat. Commun.* **10**, 3678 (2019).

63. D. L. Rocca, K. A. Wilkinson, J. M. Henley, SUMOylation of FOXP1 regulates transcriptional repression via CtBP1 to drive dendritic morphogenesis. *Sci. Rep.* **7**, 877 (2017).

64. A. J. Valente, L. A. Maddalena, E. L. Robb, F. Moradi, J. A. Stuart, A simple ImageJ macro tool for analyzing mitochondrial network morphology in mammalian cell culture. *Acta Histochem.* **3**, 315–326 (2017).

65. D. G. Hardie, B. E. Schaffer, A. Brunet, AMPK: An energy-sensing pathway with multiple inputs and outputs. *Trends Cell Biol.* **26**, 190–201 (2016).
